# Brazilian Portuguese validation of the breast cancer-related modules of the BREAST-Q

**DOI:** 10.3389/fonc.2026.1674229

**Published:** 2026-04-06

**Authors:** Antônio Bailão Junior, Idam de Oliveira-Junior, Guilherme Novita Garcia, João Bosco Ramos Borges, Vivian Aparecida Brancaglioni, Carlos Eduardo Paiva, René Aloisio da Costa Vieira

**Affiliations:** 1 Postgraduate Program in Tocogynecology, Faculty of Medicine of Botucatu, São Paulo State University (UNESP), Botucatu, SP, Brazil; 2 Department of Mastology and Breast Reconstruction, Barretos Cancer Hospital, Barretos, SP, Brazil; 3 Postgraduate Program in Oncology, Barretos Cancer Hospital, Barretos, SP, Brazil; 4 Brazilian Society of Mastology, São Paulo Regional Unit, São Paulo, SP, Brazil; 5 Researcher Support Center, Barretos Cancer Hospital, Barretos, SP, Brazil; 6 Department of Oncologic Surgery, Mastology Division, Muriaé Cancer Hospital, Muriaé, MG, Brazil

**Keywords:** breast neoplasms, mammoplasty, mastectomy, quality of life, segmental mastectomy, surveys and questionnaires

## Abstract

**Background:**

The rising incidence of breast cancer and the growing number of survivors underscore the need for further research into quality of life. The BREAST-Q is one of the most comprehensive tools for assessing quality of life (QL) in patients undergoing breast surgery. Although translated into multiple languages, it has only been fully validated in English.

**Material and methods:**

This longitudinal study was conducted to validate and evaluate the psychometric properties of the BREAST-Q in Brazilian Portuguese. Breast cancer-related modules were assessed in 195 patients, mastectomy modules in 66 patients, breast-conserving therapy (BCT) modules in 79 patients, and reconstruction modules in 50 patients. Patient characteristics were analyzed using the mastectomy, BCT, and breast reconstruction modules, along with surgical data on complications and cosmetic outcomes. The psychometric properties of the questionnaires were evaluated for reliability using Cronbach’s *α*. Test–retest reproducibility was analyzed using the intraclass correlation coefficient (ICC). Construct validity was evaluated using Spearman’s correlation coefficient. Responsiveness to change was assessed using the Wilcoxon test. Comparisons among independent groups, performed using the Kruskal–Wallis test. Convergent and discriminant validity analysis was achieved by comparing the results with those of the BCTOS and EORTC QLQ questionnaires.

**Results:**

A total of 195 patients were recruited from the outpatient breast surgical oncology clinic. The sample was divided into five groups: mastectomy without reconstruction, mastectomy with reconstruction (short form), mastectomy with reconstruction (long form), BCT, and BCT with radiotherapy (RT). Internal reliability, measured by Cronbach’s *α*, was greater than 0.7 in most modules across all groups. Reproducibility, assessed by ICC, ranged from 0.23 to 0.97, with the lowest values observed in the expectation modules, particularly expectations regarding appearance in the long-form questionnaire. The BREAST-Q exhibited good convergent and discriminant validity, with *ρ* ≥ 0.4 (positive correlations) or *ρ* ≤ −0.4 (negative correlations). Responsiveness showed that the mastectomy without reconstruction group had worse quality of life. Comparisons among independent groups demonstrated, in the postoperative phase, highest QL in the BCT with radiotherapy group.

**Conclusion:**

The findings indicate that all breast cancer-related modules of the BREAST-Q effectively measured QL in Brazilian patients.

## 1 Introduction

Breast cancer is the most common type of cancer among women in Brazil and worldwide (1). In recent decades, radical mastectomy has been increasingly replaced by breast-conserving therapy (BCT), whose indications have expanded with the advent of oncoplastic surgery. Similarly, delayed breast reconstruction using autologous flaps has gradually given way to immediate reconstruction with implants, whereas axillary lymphadenectomy has been replaced by sentinel lymph node biopsy, or, in selected cases, by omitting the axillary approach altogether. In parallel, large-scale mammographic screening and advances in systemic therapies (chemotherapy, hormone therapy, immunotherapy, and targeted therapy) and locoregional therapies (radiotherapy) have contributed to lower recurrence rates and improved survival, resulting in a growing number of breast cancer survivors. These developments prompt reflection on the quality of life of these patients and the treatment-related sequelae (2).

Quality of life is defined as an individual’s perception of their position in life within the culture and value systems in which they live and in relation to their goals, expectations, standards, and concerns (3). Several questionnaires have been developed to evaluate quality of life in the various contexts that permeate breast cancer treatment, including general instruments, breast cancer-specific questionnaires, instruments focused on breast surgery, and tools designed to assess shoulder-related sequelae, anxiety, or depression (4, 5).

The BREAST-Q is one of the key instruments used to assess the impact of breast surgery (4, 5). Developed in 2009 in the United States, the questionnaire is now in its second version (6). This modular instrument covers multiple domains of breast treatment, whether surgical or oncological. The current version has an electronic format (7) and was expanded to include specific modules related to BCT (8) and sensitivity (9). The questionnaire includes modules for mastectomy, BCT, and breast reconstruction (short- and long-forms), the latter containing specific questions for myocutaneous flaps (10, 11). Despite the relevance of the BREAST-Q, few studies have focused on validating the instrument in languages other than English (12–20). The instrument has been translated into Brazilian Portuguese (21, 22). However, only one study has evaluated some of its modules (23). To date, no validation study has been conducted on the modules specifically related to breast cancer treatment. In light of these considerations, this study aimed to validate the psychometric properties of the BREAST-Q in Brazilian Portuguese.

## 2 Material and methods

This is a prospective, cross-sectional study with the aim of validating the Brazilian Portuguese version of the BREAST-Q quality of life questionnaire. The research was conducted at the Barretos Cancer Hospital. The protocol was approved by the local Research Ethics Committee (protocol No. 1576/2018, approved on December 6, 2018). The following questionnaires were approved for use in this validation study: BREAST-Q, EORTC QLQ-C30, EORTC BR42 (24), EORTC BRECON23 (25), and BCTOS (26, 27). All modules of the BREAST-Q questionnaire had already been translated into Brazilian Portuguese, and the use of this instrument in this validation study was authorized by the authors and properly licensed by the Questionnaire Research Academy of the Memorial Sloan Kettering Cancer Center.

BREAST-Q (version 2.0) contains six main domains, including three domains related to well-being and other three related to satisfaction. Further information is available on the questionnaire’s website (https://qportfolio.org/breast-q/) and user manual webpage (https://qportfolio.org/wp-content/uploads/2018/12/BREAST-Q-USERS-GUIDE.pdf).

Patients were consecutively recruited and allocated to groups according to the type of surgery and availability. Inclusion criteria were (i) ECOG performance status 0 or 1; (ii) clinical stage 0, I, II, or III; (iii) acceptance to undergo treatment and classification into one of five pre-selected groups; and (iv) availability to complete the quality of life questionnaire. Exclusion criteria included (i) male patients with breast cancer and (ii) individuals with cognitive impairment. For the post-radiotherapy module, in addition to the above criteria, patients who had undergone BCT with adjuvant radiotherapy at least six months prior to the study were included at their first follow-up visit after treatment. This group was treated as an independent patient group. All participants provided informed consent prior to participation and were enrolled according to the calculated sample size. Information’s were reported in Tables and **Supplementary Tables (ST)**.

Sample size calculation was based on the BREAST-Q validation study (**Supplementary Table 1**) and performed separately for each questionnaire module (mastectomy, BCT, and breast reconstruction), using an *α* value ranging from 0.70 to 0.75. The final sample included 195 patients, distributed according to questionnaire modules: 66 in the mastectomy module, 79 in the BCT module (25 preoperatively and 54 post-radiotherapy), and 50 in the reconstruction module (25 for the short-form questionnaire and 25 for the long-form questionnaire). For determination of test–retest reliability (28), a minimum of 25 patients were used. The reconstruction module was analyzed in patients undergoing immediate reconstruction with implant; patients who received myocutaneous flaps were not included.

A total of 141 patients were selected in the preoperative period, and after the surgical approach was determined (mastectomy without reconstruction, mastectomy with reconstruction, or breast-conserving treatment), participation in the study was offered. Upon consent, participants were allocated into groups according to the proposed surgical treatment, and the corresponding group-specific questionnaires were administered by the researchers at that time. Seven days later (**Supplementary Table 2**), the BREAST-Q questionnaire was reapplied to assess test–retest reliability).

In the postoperative phase, the group-specific postoperative questionnaires were administered on postoperative day 7 (test) and reapplied on postoperative day 14 (retest). A third clinical evaluation was performed before postoperative day 30, at which time only clinical data related to possible surgical complications were collected; no quality-of-life questionnaires were administered at this visit.

The remaining 54 patients belonging to the post-radiotherapy breast-conserving treatment group (independent group), as previously mentioned, were invited to participate in the study at their first outpatient follow-up visit approximately 6 months after completion of radiotherapy. Upon consent, the questionnaires were administered at that time (test) and reapplied 7 days later (retest). The database created on the REDCap^®^ platform contains 920 variables per patient. Data collection procedures are described in **Supplementary Tables 2**–**4**. Clinical and demographic data were recorded using a standardized form.

Since the study was conducted in a Brazilian public hospital, 100% of patients were treated through the Unified Health System (SUS) protocols.

### 2.1 Statistical analysis

Data were entered into REDCap^®^ and exported to IBM^®^ SPSS^®^ for Windows^®^ version 20. Descriptive statistics were used to characterize patient groups. Two types of reliability were assessed: internal consistency (i.e., homogeneity of scales containing several items) and test–retest reliability. Internal consistency was evaluated using Cronbach’s *α*, with values >0.70 considered acceptable. Test–retest reliability was assessed using the intraclass correlation coefficient (ICC). The acceptable minimum effect index was defined as a response rate less than 5%.

For comparison with other questionnaires, convergent validity was analyzed by comparing conceptually related measures using Spearman’s correlation, with values ≥0.40 deemed acceptable. Data from the preoperative period were correlated with data from EORTC QLQ-C30 and EORTC QLQ-BR42 (24). Postoperative data were compared with EORTC QLQ-C30, EORTC QLQ-BR42 (24), EORTC QLQ-BRECON23 (25), and BCTOS data (26, 27) (**Supplementary Tables 3**, **4**). Responsiveness to change was assessed by comparing only the groups and domains that had both pre- and postoperative assessments. The Wilcoxon test was initially applied, and Cliff’s delta was used to measure effect size. Cliff’s delta was calculated using software RStudio with the *effsize* package.

Comparisons between independent groups in both the preoperative and postoperative phases were performed using the Kruskal–Wallis test. When statistical significance was observed (p < 0.05), *post hoc* analyses were conducted with Bonferroni correction.

## 3 Results

A total of 197 patients were recruited. Two patients were excluded due to changes in the proposed surgical procedure: one patient opted for mastectomy instead of BCT and one required intraoperative conversion to mastectomy without reconstruction due to the extent of the tumor. Thus, 197 patients completed the preoperative questionnaires, and 195 completed both preoperative and postoperative assessments.

The cohort of 195 patients was divided into five groups based on the surgical treatment received and the corresponding BREAST-Q modules: group 1, mastectomy without reconstruction (*n* = 66 patients); group 2, mastectomy with reconstruction (short form) (*n* = 25 patients); group 3, mastectomy with reconstruction (long form) (*n* = 25 patients); group 4, BCT (*n* = 25 patients); and group 5, BCT with radiotherapy (*n* = 54 patients).

**Table 1** and **Table 2** showed categorical variables related to patient characteristics. Approximately 33.0% of patients had complete primary education as their highest level of education. As for living arrangement, 76.6% reported living with a partner or family member. Almost half of the sample (49.7%) worked full-time. Regarding cancer status, 30.5% were newly diagnosed and eligible for up-front surgical treatment, whereas 42.2% had completed neoadjuvant chemotherapy. The mean tumor size was 44.6 mm. Only 29.9% of patients presented with early clinical stage (EC 0, I). The most frequent molecular subtype was luminal B HER2-negative (40.1%).

**Table 1:** Sociodemographic characteristics of the sample.

Category	Variable	*n*	%
Living arrangement	Alone	43	21.8%
	With partner	151	76.6%
	With others	3	1.5%
Sexually active	No	60	30.5%
	Yes	137	69.5%
Employment status	Employed full-time	98	49.7%
	Employed part-time	8	4.1%
	Homemaker	22	11.2%
	Unemployed	10	5.1%
	Retired	42	21.3%
	Other	17	8.6%
Education	Illiterate or incomplete primary education	13	6.6%
	Complete primary education	52	26.4%
	Incomplete or complete secondary education	75	38.1%
	Higher education	57	28.9%
Menopausal status	Premenopausal	66	33.5%
	Postmenopausal	115	58.4%
	Treatment-induced menopausal	15	7.6%
	Unknown	1	0.5%

**Table 2:** Clinical characteristics of the sample.

Category	Variable	*n*	%
Cancer status	Recently diagnosed, no chemotherapy	60	30.5
	Recently diagnosed, undergoing chemotherapy	83	42.1
	History of surgery and radiotherapy	54	27.4
Clinical stage	0	29	14.7
	I	30	15.2
	II	72	36.5
	III	66	33.5
	IV	0	0
Molecular subtype	Ductal carcinoma in situ	29	14.7
	Luminal A	32	16.2
	Luminal B HER2-negative	79	40.1
	Luminal B HER2-positive	17	8.6
	HER2-enriched	17	9.5
	Triple-negative	22	11.2
Chemotherapy	No	74	37.6
	Yes	123	62.4
	Neoadjuvant	96	78.0
	Adjuvant	27	22.0
Chemotherapy regimen	AC-T	104	84.6
	T-AC	17	13.8
	DC X 6	2	1.6
Targeted therapy	Yes	30	15.2
	No	167	84.8
Targeted therapy regimen	Trastuzumab	27	90
	Pertuzumab	1	3.3
	T-DM1	0	0
	Trastuzumab + pertuzumab	2	6.6
Breast Surgery (*n* = 197)	Oncoplastic	36	18.2
	Reconstructive	50	25.4
	No oncoplastic surgery	111	56.3
*Axillary* Surgery	Sentinel lymph node biopsy (SNLB)	104	52.8
	SNLB + axillary lymph node dissection	36	18.3
	Axillary lymph node dissection	43	21.8
	No axillary surgery	14	7.1
	Yes	18	9.1
	No	179	90.9

In the analyzed sample, 44% of patients underwent some type of oncoplastic or reconstructive breast surgery. Symmetrization was performed in only 18 patients (9.2%). Regarding axillary surgery, 52.8% of patients underwent sentinel lymph node biopsy only, 40.1% underwent axillary lymphadenectomy, and 7.1% did not undergo any axillary intervention. Since the study was conducted in a Brazilian public hospital, 100% of patients were treated through the Unified Health System (SUS) protocols.

In the present study, 51 patients (26%) experienced some type of postoperative complication, with seroma being the most common, affecting 21 patients (10.8%), followed by wound dehiscence, observed in 19 patients (9.7%). The highest rate of complications was observed in the mastectomy with immediate reconstruction group, in which 17 of 50 patients (34%) experienced complications. Wound dehiscence was the predominant complication, and two patients (4%) in this group experienced implant loss.

Analysis of continuous variables revealed that age (mean ± standard deviation) varied among groups. Patients who underwent breast reconstruction were younger than patients who did not undergo reconstruction: 44 ± 12 years in the mastectomy with reconstruction group (long form), 46 ± 10 years in the mastectomy with reconstruction group (short form), 54 ± 10 years in the BCT with radiotherapy group, and 56 ± 12 years in both the BCT without radiotherapy and the mastectomy without reconstruction groups.

Tumor size influenced surgical treatment decisions, with mean tumor sizes of 59 ± 30 mm in the mastectomy without reconstruction group, 51 ± 46 mm in the mastectomy with reconstruction group, 34 ± 33 mm in the BCT with oncoplasty group, 24 ± 15 mm in the BCT with radiotherapy group.

### 3.1 Questionnaire administration

The questionnaires were administered by research coordinators from the local Research Support Center (98 patients), researchers from the Department of Mastology and Breast Reconstruction (54 patients), and medical researchers or interns (43 patients).

In the preoperative phase, the administration of the EORTC QLQ-C30, EORTC QLQ-BR42, and BREAST-Q had a mean duration of 35 min. For groups undergoing immediate reconstruction, the mean durations were 36 min (short form) and 39 min (long form). In the postoperative period, the administration of the EORTC QLQ-C30, EORTC QLQ-BR42, BREAST-Q, BCTOS, and EORTC QLQ-BRECON23 questionnaires took on average 34 min. In the immediate reconstruction groups, the mean duration was 36 min.

### 3.2 Acceptability of the BREAST-Q

The BREAST-Q demonstrated good acceptability; 100% of patients (n = 195) included in the study completed the questionnaires, with 141 responding in both the preoperative and postoperative periods and 54 (breast-conserving treatment with radiotherapy) completing only the postoperative assessment, with a low rate of missing responses (**Supplementary Tables 5**, **6**). Most domains had response rates above 98%, with the lowest observed in the sexual well-being domain during the postoperative phase for the mastectomy with reconstruction (short form) group (76.6%). Sixty-eight patients (34.9%) required assistance with reading or completing the modules, and only five patients (2.5%) reported finding some questions confusing or difficult to answer.

### 3.3 Reliability

The internal consistency of BREAST-Q modules was evaluated using Cronbach’s *α* (**Supplementary Table 7**), which demonstrated strong reliability in both pre- and postoperative tests and retests, with *α* values above 0.8 when assessed as a whole (without separating the questionnaire into scales or domains).

Reliability was further assessed by calculating Cronbach’s *α* values for each individual domain. In the BCT module (**Supplementary Table 8**), Cronbach’s *α* exceeded 0.7 across all well-being domains in both preoperative and postoperative periods. Among the satisfaction domains, only satisfaction with breasts reached robust *α* values, ranging from 0.77 preoperatively to 0.93 postoperatively. Lower reliability was observed for satisfaction with care, with *α* values ranging from 0.12 to 0.52. This finding may be attributed to the subjective nature of satisfaction with care and to the heterogeneity of experiences among patients treated within the Brazilian Unified Health System (SUS), who receive care from multiple professionals throughout their treatment trajectory. In the validation study of the BREAST-Q conducted in Nigeria, Cronbach’s alpha values for some satisfaction domains were moderate or could not be calculated due to limited variability. These findings suggest that the observed results reflect characteristics of the study population, healthcare delivery model, and cultural context rather than intrinsic shortcomings of the instrument (19). In the BCT with radiotherapy module (**Supplementary Table 9**), high reliability was obtained across all domains, with *α* values between 0.81 and 0.95.

In the reconstruction (long form) module (**Supplementary Table 10**), all well-being domains showed *α* values above 0.7, except physical well-being, which achieved *α* values of 0.62 preoperatively and 0.53 postoperatively. Satisfaction with breasts showed strong internal consistency, with *α* values of 0.84 and 0.96 pre- and postoperatively, respectively. In the satisfaction with care domain, the highest reliability was found in the surgeon information subscale (*α* = 0.76). For the reconstruction (short form) module (**Supplementary Table 11**), reliability was excellent across all domains of well-being, satisfaction with care, and satisfaction with outcome, with *α* values above 0.8. The only exception was the postoperative physical well-being domain, which had a reasonably acceptable *α* value of 0.77.

The expectations domain for reconstruction (short form) showed adequate reliability, with *α* values above 0.8 in all scales. For the expectations domain in the reconstruction (long form) module, Cronbach’s *α* exceeded 0.8 for pain and coping, was 0.6 for appearance, and fell below 0.5 for implant and medical staff scales (**Supplementary Table 9**). In the mastectomy without reconstruction module (**Supplementary Table 12**), reliability was high across most domains, with *α* values above 0.8, except for the preoperative satisfaction with breasts domain, which had a low *α* of 0.67.

### 3.4 Test–retest reliability

The test–retest reliability of the BREAST-Q refers to the ability of the instrument to provide consistent results when administered to the same individual at different times, such as in test and retest phases. The higher the test–retest correlation of a quality of life questionnaire, the more reliable it is.

In the present study, ICC values were generally good, exceeding 0.7 in most domains across all modules. Although overall correlation was strong, some modules exhibited wide confidence intervals. The lowest ICCs were observed in the postoperative satisfaction with breasts domain, with values of 0.12 in the BCT module and 0.36 in the reconstruction (long form) module. **Supplementary Table 13** summarizes the ICC values for major domains across all groups.

In short and long forms of breast reconstruction (**Supplementary Table 13**), most scales demonstrated good reliability, with ICCs above 0.7. The only exception was the expectations for appearance scale in the long form, which had a low ICC of 0.23 (**Supplementary Table 14**).

### 3.5 Construct validity

Construct validity was assessed through convergent and discriminant validation of the BREAST-Q by comparing it with questionnaires already validated in Brazilian Portuguese. In the preoperative phase, the instrument was compared with EORTC QLQ-C30 and EORTC QLQ-BR42. In the postoperative phase, it was compared with EORTC QLQ-C30 and EORTC QLQ-BR42 in all groups, as well as with EORTC QLQ-BRECON23 in the immediate reconstruction group and BCTOS in the BCT groups. Mean questionnaire scores by treatment group are presented in **Supplementary Tables 15**, **16**.

In the BCT module during the preoperative phase (**Table 3**), Spearman correlation coefficients (*ρ*) exceeded 0.4 for positive associations and were below −0.4 for negative associations. It is noteworthy that the satisfaction with breasts domain did not reach the expected thresholds; however, in the postoperative phase, all domains showed good construct validity (**Table 4**).

**Table 3:** Construct validity* of the breast-conserving therapy module in the preoperative phase (*n* = 25 patients).

BREAST-Q			Well-being			Satisfaction		
			Physical	Psychosocial	Sexual	Breast	Outcome	Care
QLQ-C30	Global health		0.12	0.49	0.30	0.28	–	–
	Function scales	Physical	0.20	–	–	–	–	–
		Role	0.13	–	–	–	–	–
		Emotional	–	0.45	0.57	–	–	–
		Cognitive	–	0.16		–	–	–
	Symptom scale	Pain	−0.40	–	–	–	–	–
QLQ-BR42	Function scales	Body image	0.14	0.44	0.24	0.16	–	–
		Sexual functioning	–	–	0.65	–	–	–
		Sexual enjoyment	–	–	0.68	−0.32	–	–
	Symptom scales	Systemic therapy side effects	0.07	–	–	–	–	–
		Breast symptoms	−0.48	–	–	−0.26	–	–
		Arm symptoms	−0.34	–	–	–	–	–
		Endocrine symptoms	0.04*	–	–	–	–	–

*Spearman's correlation.

**Table 4:** Construct validity* of the breast-conserving therapy module in the postoperative phase (*n* = 25 patients).

BREAST-Q			Well-being			Satisfaction		
			Physical	Psychosocial	Sexual	Breast	Outcome	Care
QLQ-C30	Global health		0.34	0.46	0.40	0.38	–	
	Function scales	Physical	0.30	–	–	–	–	–
		Role	0.28	–	–	–	–	–
		Emotional	–	0.35	0.34	–	–	–
		Social	–	0.24	–	–	–	–
	Symptom scales	Pain	−0.60	–	–	–	–	–
		Financial difficulties	–	–	–	–	–	–
QLQ-BR42	Function scales	Body image	0.16	0.57	0.48	0.46	–	–
		Sexual functioning	–	–	0.52	–	–	
		Sexual enjoyment	–	–	0.62	0.37	–	
		Satisfaction with breast in the postoperative phase	–	–	–	–	–	
	Symptom scales	Systemic therapy side effects	−0.27	–	–	–	–	
		Breast symptoms	−0.55	–	–	−0.51	–	
		Arm symptoms	−0.45	–	–	–	–	
		Endocrine symptoms	−0.22	–	–	–	–	
BCTOS		Functional status	−0.45	–	–	–	–	
		Cosmetic status	–	–	–	–	−0.49	
		Breast sensitivity status	–	–	–	–	−0.70	
		Edema status	−0.20	–	–	–	–	

*Spearman's correlation.

For the BCT with radiotherapy module, correlation coefficients in the postoperative phase were above the predefined thresholds. A *ρ* of 0.44 was found for the correlation between adverse effects of radiation, which is specific to this group, and EORTC QLQ-C30 global health (**Table 5**).

**Table 5:** Construct validity* of the breast-conserving therapy with radiotherapy module in the postoperative period (*n* = 54 patients).

BREAST-Q			Well-being			Satisfaction		
			Physical	Psychosocial	Sexual	Breast	Outcome	Adverse effects of radiation
QLQ-C30	Global health		0.36	0.35	0.22	0.31	–	0.44
	Function scales	Physical	0.28	–	–	–	–	–
		Role	0.35	–	–	–	–	0.21
		Emotional	–	0.32	0.20	–	–	–
		Social	–	0.09	–	–	–	–
	Symptom scale	Pain	−0.60	–	–	–	–	–
QLQ-BR42	Function scales	Body image	0.34	0.43	0.41	0.46	0.44	0.24
		Sexual functioning	–	–	0.60	–	–	–
		Sexual enjoyment	–	–	0.62	0.37	0.21	–
	Symptom scales	Systemic therapy side effects	−0.26	–	–	–	–	
		Breast symptoms	−0.53	–	–	−0.51	0.40	
		Arm symptoms	−0.35	–	–	–	–	
		Endocrine symptoms	−0.30	–	–	–	–	
BCTOS		Functional status	−0.40	–	–	–	–	
		Cosmetic status	–	–	–	–	−0.65	
		Breast sensitivity status	–	–	–	–	−0.50	

*Spearman's correlation.

For the reconstruction (short form) module in the preoperative phase (**Table 6**), a strong inverse correlation was observed between physical well-being and breast symptoms (*ρ* = −0.87). However, correlations for psychosocial well-being and satisfaction with breasts were lower than expected. In the postoperative phase (**Table 7**), all domains showed adequate correlations with their respective scales or subscales from other questionnaires. Notably, satisfaction with breasts correlated strongly with the corresponding EORTC QLQ-BRECON-23 scale (*ρ* = 0.84).

**Table 6:** Construct validity* of the reconstruction (short form) module in the preoperative period (*n* = 25 patients).

BREAST-Q			Physical	Psychosocial	Sexual	Breast	Outcome	Care
QLQ-C30	Global health		0.32	0.01	0.01	0.40	–	–
	Function scales	Physical	0.20	–	–	–	–	–
		Role	0.16	–	–	–	–	–
		Emotional	–	0.36	0.05	–	–	–
		Cognitive	–	0.16		–	–	–
		Social	–	0.05		–	–	–
	Symptom scale	Pain	−0.47	–	–	–	–	–
QLQ-BR42	Function scales	Body image	0.13	0.03	0.20	0.13	–	–
		Sexual functioning	–	–	0.23	–	–	–
		Sexual enjoyment	–	–	0.40	0.08	–	–
	Symptom scales	Systemic treatment side effects	0.38	–	–	–	–	–
		Breast symptoms	−0.87	–	–	−0.19	–	–
		Arm symptoms	−0.46	–	–	–	–	–
		Endocrine symptoms	0.22	–	–	–	–	–

*Spearman's correlation.

**Table 7:** Construct validity* of the reconstruction (short form) module in the postoperative period (*n* = 25 patients).

BREAST-Q			Well-being			Satisfaction		
			Physical	Psychosocial	Sexual	Breast	Outcome	Care
QLQ-C30	Global health		0.05	0.30	0.44	0.26	0.43	
	Function scales	Physical	0.55	–	–	–	–	–
		Role	0.23	–	–	–	0.28	–
		Emotional	–	0.20	0.03	–	–	–
		Social	–	0.14	–	–	–	–
	Symptom scale	Pain	−0.59	–	–	–	–	–
QLQ-BR42	Function scales	Body image	0.03	0.42	0.35	0.55	0.33	–
		Sexual functioning	–	–	0.62	–	–	
		Sexual enjoyment	–	–	0.60	0.39	–	
	Symptom scales	Systemic therapy side effects	−0.37	–	–	–	–	
		Breast symptoms	−0.47	–	–	−0.09	–	
		Arm symptoms	−0.41	–	–	–	–	
		Endocrine symptoms	−0.32	–	–	–	–	
BRECON23		Treatment side effects	−0.14	–	–	–	–	
		Loss of nipple	–	–	–	−0.58	–	
		Sexual functioning	–	–	0.43	–	–	
		Satisfaction with breasts	–	0.73	–	0.84	–	
		Satisfaction with nipple	–	0.41	–	0.50	–	
		Satisfaction with surgery	–	0.60	–	0.72	–	
		Nipple preservation	–	–	–	0.31	−0.39	

*Spearman's correlation.

The reconstruction (long form) pre-operative module (**Table 8**) showed correlation values consistent with the study’s validation criteria, both pre- and postoperatively, with *ρ* ≥ 0.4 for positive associations and *ρ* ≤ −0.4 for negative associations. In the postoperative phase, strong correlations were found between BREAST-Q domains and equivalent EORTC QLQ-BRECON-23 subscales (**Table 9**).

**Table 8:** Construct validity* of the reconstruction (long form) module in the preoperative period (*n* = 25 patients).

BREAST-Q			Well-being			Satisfaction		
			Physical	Psychosocial	Sexual	Breast	Outcome	Care
QLQ-C30	Global health		0.23	0.08	0.07*	0.03*	–	–
	Function scales	Physical	0.16	–	–	–	–	–
		Role	−0.05	–	−01.8	–	–	–
		Emotional	–	0.46		–	–	–
		Cognitive	–	0.22		–	–	–
		Social	–	0.04		–	–	–
	Symptom scale	Pain	−0.36	–	–	–	–	–
QLQ-BR42	Function scales	Sexual functioning	–	–	0.42	–	–	–
		Sexual enjoyment	–	–	0.20	0.01	–	–
	Symptom scales	Systemic therapy side effects	0.07	–	–	–	–	–
		Breast symptoms	−0.61	–	–	0.001	–	–
		Arm symptoms	−0.58	–	–	–	–	–
		Endocrine symptoms	0.11	–	–	–	–	–

*Spearman's correlation.

**Table 9:** Construct validity* of the reconstruction (long form) module in the postoperative period (*n* = 25 patients).

BREAST-Q			Well-being			Satisfaction		
			Physical	Psychosocial	Sexual	Breast	Outcome	Care
QLQ-C30	Global health		0.47	0.47	0.40	0.15	0.36	
	Function scales	Physical	0.50	–	–	–	–	–
		Role	0.12	–	0.20	–	0.03	–
		Emotional	–	0.32		–	–	–
		Social	–	−0.18	–	–	–	–
	Symptom scale	Pain	−0.60	–	–	–	–	–
QLQ-BR42	Function scales	Body image	0.14	0.53	0.51	0.56	0.55	–
		Sexual functioning	–	–	0.30	–	–	–
		Sexual enjoyment	–	–	0.44	−0.17	–	–
	Symptom scales	Systemic therapy side effects	−0.18	–	–	–	–	–
		Breast symptoms	−0.70	–	–	0.14	–	–
		Arm symptoms	−0.31	–	–	–	–	–
		Endocrine symptoms	−0.01	–	–	–	–	–
BRECON23		Treatment side effects	0.19	–	–	–	–	–
		Loss of nipple	–	–	–		0.12	–
		Sexual functioning	–		0.77	–	–	–
		Satisfaction with breasts	–	0.56	–	0.53	–	–
		Satisfaction with nipple	–	0.29	–	0.16	–	–
		Satisfaction with surgery	–	0.64	–	0.52	–	–
		Nipple preservation	–	–	–	−0.30	0.30	–

*Spearman's correlation.

Finally, convergent and discriminant validity analysis of the mastectomy module showed satisfactory results in both pre- and postoperative periods (**Tables 10**, **11**). In particular, the postoperative period showed strong correlations between the psychosocial well-being and satisfaction with breasts domains of BREAST-Q and the body image subscale of EORTC QLQ-BR42, with *ρ* = 0.76 and *ρ* = 0.71, respectively.

**Table 10:** Construct validity* of the mastectomy without reconstruction module in the preoperative period (*n* = 66 patients).

BREAST-Q			Well-being			Satisfaction		
			Physical	Psychosocial	Sexual	Breast	Outcome	Care
QLQ-C30	Global health		0.60	0.55	0.41	0.22*	–	–
	Function scales	Physical	0.23	–	–	–	–	–
		Role	0.52	–	0.29	–	–	–
		Emotional	–	0.47		–	–	–
		Social	–	0.45	–	–	–	–
	Symptom scale	Pain	−0.56	–	–	–	–	–
QLQ-BR42	Function scales	Body image	0.49	0.51	0.26	0.41	–	–
		Sexual functioning	–	–	0.46	–	–	–
		Sexual enjoyment	–	–	0.57	−0.18	–	–
	Symptom scales	Systemic therapy side effects	−0.38	–	–	–	–	–
		Breast symptoms	−0.42	–	–	−0.41	–	–
		Arm symptoms	−0.48	–	–	–	–	–
		Endocrine symptoms	−018	-	-	-	-	-

*Spearman's correlation.

**Table 11:** Construct validity* of the mastectomy without reconstruction module in the postoperative period (*n* = 66 patients).

BREAST-Q			Well-being			Satisfaction		
			Physical	Psychosocial	Sexual	Breast	Outcome	Care
QLQ-C30	Global health		0.55	0.80	0.57	0.68	–	
	Function scales	Physical	0.44	–	–	–	–	–
		Role	0.29	–	–	–	–	–
		Emotional	–	0.60	0.53	–	–	–
		Social	–	0.37	–	–	–	–
	Symptom scale	Pain	−0.60	–	–	–	–	–
QLQ-BR42	Function scales	Body image	0.1	0.76	0.25	0.71	–	–
		Sexual functioning	–	–	0.25	−0.10	–	
		Sexual enjoyment	–	–	0.64		–	
	Symptom scales	Systemic therapy side effects	−0.27	–	–	–	–	
		Breast symptoms	−0.55	–	–	−0.43	–	
		Arm symptoms	−0.48	–	–	–	–	
		Endocrine symptoms	−0.22	-	-	-	-	

*Spearman's correlation.

### 3.6 Responsiveness to changes

Responsiveness to change, assessed by comparing the same patients in the pre- and postoperative periods, demonstrated a decline in BREAST-Q scores across all groups. However, the group undergoing mastectomy without reconstruction exhibited the poorest overall performance. Breast reconstruction, as well as breast-conserving treatment, attenuated the negative impact of treatment on the psychosocial and sexual domains, with p values > 0.05, indicating no statistically significant differences between the pre- and postoperative phases in these groups (**Table 12**). In addition, Cliff’s delta (δ) values below 0.17 in these domains indicate a small or negligible treatment effect.

**Table 12:** Responsiveness to change from the pre- to the postoperative period.

Module	Scale	Preoperative median	Postoperative median	Z	p-value	Cliff’s delta (IC 95%) δ	Interpretation
Breast-conserving therapy	Psychosocial	72	71	-0.33	0.73	0.03 (-0.27 – 0.33)	Negligible effect
	Sexual	62	66	-0.70	0.94	-0.06 (-0.39 – 0.28)	Negligible effect
	Satisfaction with breasts	64	25	-4.11	0.00	1.00 (0.99-1.00)	Important worsening
	Physical	80	73	-2.51	0.01	0.31 (-0.01 – 0.56)	Small effect
Reconstruction (short and long form)	Psychosocial	66	62	-1.89	0.06	0.17 (0.05-0.38)	Small effect
	Sexual	66	56	-0.48	0.62	0.08 (-0.16-0.32)	Negligible effect
	Satisfaction with breasts	71	53	-5.19	0.00	0.74 (0.56 −0.84)	Important worsening
	Physical	96	68	-5.46	0.00	0.71 (0.50 −0.84)	Important worsening
Mastectomy without reconstruction	Psychosocial	69	39	-5.56	0.00	0.53 (0.34 – 0.67)	Important worsening
	Sexual	50	39	-5.34	0.00	0.47 (0.27 – 0.63)	Moderate effect worsening
	Satisfaction with breasts	64	34	-5.94	0.00	0.68 (0.51 – 0.79)	Important worsening
	Physical	85	64	-4.37	0.00	0.48 (0.27 – 0.64)	Important worsening

Interpretation of Cliff’s delta (δ): |δ| < 0.147: negligible effect; 0.147 ≤ |δ| < 0.33: small effect; 0.33 ≤ |δ| < 0.474: moderate effect; |δ| ≥ 0.474: large effect.

The physical well-being domain showed a significant decline in the mastectomy without reconstruction and mastectomy with reconstruction groups (p < 0.05), with Cliff’s delta values above 0.48, indicating a large treatment effect. The breast-conserving treatment group also demonstrated a decline in this domain; however, the magnitude of the effect was small (δ = 0.31). The satisfaction with breasts domain showed a significant decline in all groups (p < 0.05), with a large magnitude of effect, as indicated by δ values above 0.68 (**Table 12**).

### 3.7 Comparison between independent groups

The comparison between independent groups demonstrated that, in the preoperative phase (**Table 13**), there were no statistically significant differences among groups across all well-being domains and breast satisfaction, as all p-values were greater than 0.05 (**Table 13**).

**Table 13:** Preoperative comparison between independent groups.

Domain	n	H	Global p-value	Comparison between groups	Adjust p-value
Physical well-being: chest	141	5,58	0,061	1vs2 1vs4 2vs4	>0,99 0,06 0,35
Psychosocial well-being	141	2,05	0,360	1vs2 1vs4 2vs4	0,49 >0,99 0,68
Sexual well-being	141	1,50	0,473	1vs2 1vs4 2vs4	>0,99 0,663 >0,999
Satisfaction with breasts	141	2,00	0,367	1vs2 1vs4 2vs4	>0,99 0,60 0,79

Group 1, mastectomy without reconstruction (N = 66); Group 2, breast-conserving treatment (N = 25); Group 4, mastectomy with reconstruction (N = 50).

In the postoperative phase (**Table 14**), comparisons among groups demonstrated statistically significant differences across several domains.

**Table 14:** Postoperative comparison between independent groups.

Domain	n	H	Global p-value	Comparison between groups	Adjust p-value
Physical well-being: chest	195	18,41	0,023 0,000 0,738 0,485 0,054 0,001	1vs2 1vs3 1vs4 2vs3 2vs4 3vs4	0,136 0,001 1,000 1,000 0,326 0,008
Psychosocial well-being	195	41,74	0,000 0,000 0,000 0,912 0,169 0,067	1vs2 1vs3 1vs4 2vs3 2vs4 3vs4	0,000 0,000 0,000 1,000 1,000 0,402
Sexual well-being	195	43,61	0,000 0,000 0,000 0,851 0,696 0,787	1vs2 1vs3 1vs4 2vs3 2vs4 3vs4	0,000 0,000 0,000 1,000 1,000 1,000
Satisfaction with breasts	195	95,79	0,023 0,000 0,002 0,000 0,000 0,000	1vs2 1vs3 1vs4 2vs3 2vs4 3vs4	0,139 0,000 0,012 0,000 0,000 0,000

Group 1, mastectomy without reconstruction (N = 66); Group 2, breast-conserving treatment (N = 25); Group 3, breast-conserving treatment with radiotherapy (N = 54); Group 4, mastectomy with reconstruction (N = 50).

For the physical well-being domain, significant differences were observed (adjusted *p* < 0.05) between the mastectomy without reconstruction and breast-conserving treatment with radiotherapy groups, as well as between the immediate breast reconstruction and breast-conserving treatment with radiotherapy groups. Patients undergoing breast-conserving treatment with radiotherapy achieved the highest scores.

In the psychosocial well-being and sexual well-being domains, significant differences (adjusted *p* < 0.001) were observed between the mastectomy without reconstruction group and all other groups (breast-conserving treatment without radiotherapy, immediate breast reconstruction, and breast-conserving treatment with radiotherapy). In contrast, no differences were observed among the remaining groups, indicating that patients undergoing breast reconstruction and breast-conserving treatment had comparable and superior quality of life in these domains compared with those undergoing mastectomy without reconstruction (**Table 14**).

Evaluating satisfaction with breasts domain, significant differences (adjusted *p* < 0.05) were observed between the mastectomy without reconstruction and breast-conserving treatment with radiotherapy groups, as well as between the mastectomy without reconstruction and immediate breast reconstruction groups. Additionally, the breast-conserving treatment with radiotherapy group differed from all other groups (mastectomy without reconstruction, breast-conserving treatment without radiotherapy, and immediate breast reconstruction), presenting the highest BREAST-Q scores for this domain.

Finally, the satisfaction with care domain showed no statistically significant differences among groups (**Table 15**).

**Table 15:** Comparison between independent groups for satisfaction with care.

Domain	n**	H	p-value	Significance
Satisfaction with surgeon	141	4.72	0,094	ws
Satisfaction with medical team	141	4.63	0,099	ws
Satisfaction with office staff	141	2,043	0,360	ws

**The breast-conserving treatment with radiotherapy group was excluded from this analysis, as it represents an independent late postoperative group. ws, without significance.

## 4 Discussion

As patients’ clinical outcomes of recurrence and survival continue to improve and the introduction of more conservative and cosmetic surgical techniques, quality of life assessment has become a fundamental part of the surgical decision-making process. Outcome analysis now extends beyond the final breast shape to include satisfaction with cosmetic results, residual sensitivity, treatment-related sequelae, and body image. The literature on quality of life in patients undergoing surgical treatment for breast cancer is conflicting. A U.S. study based on SEER data from Detroit and Los Angeles supports that aesthetic satisfaction among patients undergoing breast-conserving treatment is similar to that of those who underwent mastectomy with reconstruction (29), whereas another study conducted at the Memorial Sloan Kettering Cancer Center demonstrated that patients treated with breast-conserving surgery had higher quality-of-life scores compared with those who underwent mastectomy with reconstruction, from the immediate postoperative period up to 3 years after surgery (30). Therefore, specific instruments are needed to assess these dimensions (5), and the BREAST-Q remains one of the main questionnaires available for use in breast cancer treatment (10).

The interest in validating the BREAST-Q for Brazilian Portuguese arose from the need for more comprehensive instruments capable of assessing quality of life across all its physical and psychosocial dimensions, given that breast cancer surgeries impact physical, sexual, and social dimensions (31). Measuring quality of life in breast cancer patients undergoing surgical treatment is essential, as the impact on self-image caused by breast loss can influence a patient’s decision to pursue BCT rather than mastectomy (32). Furthermore, objectively assessing satisfaction with care is critically important, as this factor may serve as an independent predictor of survival in breast cancer (33).

The validation of quality of life instruments entails a complex methodological process, including assessments of internal consistency, stability, reproducibility, and cross-cultural equivalence. These steps are necessary for comparisons between different clinical and cultural contexts. In the case of BREAST-Q, this process is particularly complex because of the questionnaire’s length, the specificities of surgical modules, and variations between pre- and postoperative versions. These factors make it difficult to standardize validation studies. With the aim of expanding the clinical applicability of the instrument in Brazilian Portuguese, this study sought to validate, in an integrated way, the surgical modules related to breast cancer, addressing their specificities and separately analyzing the domains and indications.

This study has a potential selection bias, given that patients with larger tumors were predominantly subjected to mastectomy without reconstruction, whereas younger women with intermediate tumors were subjected to mastectomy with reconstruction. However, as this is a psychometric validation study and not a comparison between surgical groups, this heterogeneity does not compromise the objectives, which are centered on the evaluation of the metric properties of the instrument in its different clinical applications.

### 4.1 BCT module

BCT is the most common therapeutic modality for the treatment of breast cancer, particularly in the early stages of the disease, as it has positive impact on the quality of life of patients (3). The reliability of the BCT without radiotherapy module was high for all domains of well-being and satisfaction in the preoperative period, with Cronbach’s *α* values above 0.77. In Brazil, Schunk et al. (13) observed a similar reliability (*α* > 0.8) for all domains in a cohort of 58 patients. Stolpner et al. (31), in a German study encompassing 253 patients in the preoperative period, reported similar values (13, 31). For the postoperative period, the well-being domains also exhibited excellent reliability values (*α* > 0.86). However, reliability varied markedly between satisfaction domains. Evaluating our numbers, the reliability of satisfaction with breasts was excellent (*α* = 0.93), but the satisfaction with office staff was poor (*α* = 0.12). The low Cronbach’s *α* value may be attributed to various factors, including an insufficient number of items, low correlation between items, item heterogeneity, measurement errors, reverse scoring, sample size, problems with the sample population, or inconsistencies in item formulation. The hypothesis of possible failures in scale structure (item insufficiency, low correlation between items, heterogeneity) can be disregarded because the BREAST-Q BCT module has been validated in several countries, such as Germany and Malaysia (13, 17, 31).

The test–retest reliability of the BCT module was considered strong for all domains in the preoperative period, with ICC values of 0.72 to 0.87. These results are similar to those of other international validation studies. In Finland, the ICC ranged from 0.79 to 0.89 and, in Malaysia, it ranged from 0.59 to 0.90 (17, 20). For the postoperative period, the reliability of the BCT module was strong to moderate, with ICC values of 0.54 to 0.94 (20).

For construct validation, BREAST-Q was compared with other questionnaires. The physical well-being domain for the preoperative period of the BCT module showed an adequate negative correlation with the EORTC QLQ-C30 pain subscale (*ρ* = −0.40) and EORTC QLQ-BR42 breast symptoms (*ρ* = −0.48). Psychosocial well-being was moderately correlated with the EORTC QLQ-C30 scales global health (*ρ* = 0.49) and emotional functioning (*ρ* = 0.45) and EORTC QLQ-BR42 body image subscale (*ρ* = 0.44). The sexual well-being domain showed a strong correlation with EORTC QLQ-C30 emotional functioning (*ρ* = 0.45) and EORTC QLQ-BR42 sexual functioning (*ρ* = 0.65) and sexual enjoyment (*ρ* = 0.68). The German validation study for the BCT module obtained similar correlation values for physical and psychosocial well-being domains, demonstrating that these domains measure the same construct (34). Here, for the postoperative BCT module, there were significant correlations between physical, sexual, and psychosocial well-being domains and satisfaction with breasts. The correlations were considered moderate to strong compared with correlated scales of other questionnaires. There was a strong negative correlation between satisfaction with outcome and breast pain symptoms (BCTOS) (*ρ* = −0.7).

The BCT with radiotherapy module exhibited high internal reliability (Cronbach’s *α* = 0.81–0.94) and excellent test–retest reliability for well-being domains and most satisfaction domains (ICC = 0.85). Convergent and discriminant validity was achieved, with adequate correlation coefficients (*ρ* ≥ 0.4).

Previous validation studies of the BREAST-Q BCT module differ from the present study in several aspects. Some addressed only the postoperative period, whereas others evaluated both pre- and postoperative periods. Furthermore, the instrument was not applied to the same patients; instead, independent samples were used, precluding analysis of the responsiveness to change within a single study. Additionally, prior studies did not demonstrate the questionnaire’s sensitivity through known-group comparisons (17, 20, 31). Some studies present methodological limitations, such as a very short interval between the surgical intervention and questionnaire administration, or failure to report the time elapsed between surgery and questionnaire application. Additionally, the absence of data regarding the type of axillary surgery is a relevant limitation, as axillary lymphadenectomy is an independent predictor of worse quality of life (35).

### 4.2 Reconstruction module

Measuring the impact of immediate breast reconstruction can support both pre- and postoperative management. In the preoperative period, it enables the identification of patients who require more detailed information about the cosmetic outcomes of reconstructive surgery. This facilitates realistic expectation setting, reinforces the therapeutic plan, and helps prevent frustration due to mismatched expectations. In the postoperative period, this assessment contributes significantly to the patient’s acceptance of their new body image following mastectomy, with a positive impact on quality of life (16, 36). In this study, patients who underwent mastectomy with reconstruction were predominantly younger women, with a mean age of 44 years; the youngest participant was only 23 years old, representing a bias selection.

The reconstruction (short form) module had good internal reliability, with a Cronbach’s *α* above 0.77 for all well-being and satisfaction domains. Test–retest reliability was adequate, with ICC values greater than 0.72. The worst CI was observed in satisfaction with office staff (95% CI = 0.38–0.88). These findings are consistent with international validation studies conducted in Malaysia and Finland, which demonstrated satisfactory levels of instrument reliability and reproducibility across distinct cultural contexts (17, 20).

The reconstruction (long form) module exhibited good internal reliability for well-being domains in the preoperative (*α* = 0.62–0.91) and postoperative (*α* = 0.53–0.95) periods. Satisfaction with breasts showed excellent reliability (*α* > 0.84) in both pre- and postoperative periods. The decrease in Cronbach’s α values in the satisfaction domains observed in the postoperative period may be related to the high complication rate, which reached 34%. Although some studies have shown that postoperative complications can negatively affect breast satisfaction scores and certain care-related satisfaction domains of the BREAST-Q questionnaire, no study has directly evaluated the impact of these complications on the instrument’s internal consistency (37).

However, this does not affect questionnaire validation, as the reliability of the reconstruction module (short form) is well established (*α* > 0.77). Additionally, studies conducted in developing countries such as Nigeria reported Cronbach’s *α* values below 0.7. Notably, the referred study validated the mastectomy without reconstruction module (19).

The test–retest reliability of the reconstruction (short form) module was high, with ICC values above 0.72 for all domains. These results are compatible with international validation studies conducted in Malaysia and Finland (17, 20). The reconstruction (long form) module received ICC values above 0.7 for all domains except satisfaction with medical team (ICC = 0.52).

The convergent and discriminant validity in the preoperative period was evaluated through the correlation between BREAST-Q domains and EORTC QLQ-C30 and EORTC QLQ BR-42 instruments. Consistent correlations were observed for both reconstruction groups, with Spearman coefficients of ≥0.4 or ≤−0.4, as shown in the comparison matrix (**Supplementary Table 3**, **Supplementary Material**). There was a strong negative correlation between physical well-being and breast symptoms (*ρ* = −0.87), moderate correlation between psychosocial well-being and emotional functioning (*ρ* = 0.45), and moderate correlation between satisfaction with breasts and body image (*ρ* = 0.44).

For analysis of validity in the postoperative period, the reconstruction module of BREAST-Q was compared with the following instruments: EORTC QLQ-C30, EORTC BR-42, and EORTC QLQ-BRECON23. Convergent and discriminant validity was observed for all domains of BREAST-Q when correlated with the scales and subscales of reference instruments (**Supplementary Table 4**, **Supplementary Material**). Notably, physical well-being had a positive correlation with physical functioning (*ρ* = 0.55) and a negative correlation with pain (*ρ* = −0.59) and breast symptoms (*ρ* = −0.47). Psychosocial well-being correlated strongly with cosmetic satisfaction (*ρ* = 0.73) and moderately with body image (*ρ* = 0.42 and *ρ* = 0.53). Sexual well-being showed a strong correlation with EORTC QLQ-BRECON23 sexual functioning (*ρ* = 0.77) and a moderate correlation with EORTC QLQ-BR42 sexual functioning (*ρ* = 0.44). There was a strong correlation between satisfaction with breasts and EORTC QLQ-BRECON23 cosmetic satisfaction (*ρ* = 0.84 and 0.53) and body image (*ρ* = 0.55). Satisfaction with outcome had a strong correlation with body image (*ρ* = 0.55).

The inclusion of EORTC QLQ-BRECON23 as a comparison tool for the postoperative reconstruction module enriched the analysis, reinforcing the convergent and discriminant validity of the majority of the evaluated domains. EORTC QLQ-BRECON23 is crucial for evaluating the quality of life of women subjected to reconstructive breast surgery (25).

Patients’ expectations directly influence their experiences and perceptions of surgery outcomes. Adequate clarification by the surgical team contributes to the alignment of expectations, promoting more individualized preoperative care and greater patient satisfaction in the postoperative period (38). Although there is no gold standard for the validation of expectations, classical psychometric measures such as internal consistency (Cronbach’s *α*) and test–retest reliability (ICC) may ensure the robustness of the construct for the target population (21).

The BREAST-Q expectations module was divided into two forms: short and long. The short form contains 5 items with 23 phrases, whereas the long form has 27 items distributed over 114 phrases. The module is grouped into four subscales, namely pain, coping, support, and appearance. Here, each expectations module (short and long forms) was applied to 25 patients in the preoperative period. The short form had excellent reliability, with an *α* of 0.86 for expectations with pain and 0.81 for expectations with appearance.

The long form achieved Cronbach’s *α* values of 0.82 for pain, 0.83 for coping, 0.62 for appearance, and 0.35 for support from medical team. The appearance subscale showed significant improvement in reliability (*α* = 0.85) with the exclusion of the item “Will I look normal when I look in the mirror?”. In the expectations of support with medical staff subscale, exclusion of the item “Will the surgeon and nurses always be available whenever I need them?” would result in an *α* of 0.5. Moreover, exclusion of the item “Will I receive medical care promptly if I need it?” would result in an *α* of 0.76. This indicates that such questions are not very well adapted to the study population and, especially in the expectation of support from the medical team. The drop in reliability could be related to the institutional care model proposed by the unified health system, in which there is no professional as a reference but rather the institution.

The test–retest reliability of the expectations domains of BREAST-Q was acceptable for both forms. The short form showed better performance, with ICC values of 0.54 to 0.88. The reliability of the long form was within acceptable thresholds, with ICC values of 0.23 to 0.96 and 0.97, as it was observed in other studies (16).

### 4.3 Mastectomy module

The mastectomy group included 66 patients. The module showed excellent reliability in all domains in both pre- and postoperative periods, with a Cronbach’s *α* greater than 0.85 for all domains, except for preoperative satisfaction with breasts (*α* = 0.67). Similar results were observed in the Nigerian validation study, although limited to the postoperative period, with a minimum *α* of 0.43 for the domain. The study conducted in Malaysia demonstrated superior internal consistency, with all domains exhibiting *α* > 0.85.

The module’s test–retest reliability was excellent, with an ICC value greater than 0.85 for the preoperative period and above 0.79 for the postoperative period. The Malaysian study reported similar ICC values, whereas the Nigerian study reported lower ICCs (0.31 to 0.64) and did not calculate the internal consistency of satisfaction with care (surgeon, medical team, and office staff) because of the low variability in responses (floor effect) (17, 19).

Convergent and discriminant validity was confirmed, with Spearman coefficients ≥0.4 or ≤−0.4, according to the correlation matrix (**Supplementary Tables 3**, **S4**, **Supplementary Material**). In the preoperative period, physical well-being had a strong positive correlation with global health (*ρ* = 0.60) and a moderate negative correlation with breast symptoms (*ρ* = −0.42) and arm symptoms (*ρ* = −0.48). Psychosocial well-being was strongly correlated with body image (*ρ* = 0.51) and moderately correlated with emotional functioning (*ρ* = 0.47). Sexual well-being had a strong correlation with sexual enjoyment (*ρ* = 0.57) and sexual functioning (*ρ* = 0.46). Satisfaction with breasts was moderately correlated with body image (*ρ* = 0.41). In the postoperative period, there was a strong correlation between physical well-being and overall health (*ρ* = 0.55) and a strong negative correlation with pain (*ρ* = −0.6) and breast symptoms (*ρ* = −0.55). Psychosocial well-being in the postoperative period correlated strongly with body image (*ρ* = 0.76) and emotional functioning (*ρ* = 0.60). Sexual well-being had a strong correlation with sexual enjoyment (*ρ* = 0.64), emotional functioning (*ρ* = 0.53), and global health (*ρ* = 0.67). Satisfaction with breasts correlated strongly with body image (*ρ* = 0.71) and global health (*ρ* = 0.68). Of note, the Nigerian study reported similar correlations, but the analysis was limited to the postoperative period (19).

### 4.4 Responsiveness to change

Pre- and postoperative responses were compared to assess the questionnaire’s responsiveness to change. Regarding quality of life, better outcomes were observed among patients undergoing breast-conserving treatment (BCT) and mastectomy with immediate reconstruction (3). Quality of life questionnaires generally compare patients with one another, without assessing differences between pre- and postoperative periods. In this validation study, changes were observed in the postoperative period. The questionnaire was administered on postoperative day 7, which may have influenced the results. Nevertheless, in agreement with the literature, scores across all domains worsened in the postoperative period among patients undergoing mastectomy without reconstruction.

In this study, comparisons among independent groups demonstrated better quality of life for patients undergoing breast-conserving treatment with radiotherapy across all well-being domains and breast satisfaction. The breast-conserving treatment without radiotherapy group exhibited quality of life comparable to that of the mastectomy with reconstruction group in the psychosocial and sexual well-being domains. Mastectomy without reconstruction was associated with the poorest overall quality of life (3). The satisfaction with care domain did not show significant differences among groups.

### 4.5 General considerations

This study has relevant particularities, as it aimed to validate the BREAST-Q for Brazilian Portuguese. The research was conducted in a public institution where all patients are treated through *Sistema Único de Saúde* (SUS). Despite the high levels of satisfaction with care—ranging from 75.7 for satisfaction with information: surgeon to 97.5 for satisfaction with office staff (**Supplementary Table 9**, **Supplementary Material**)—the institutional care model, in which patients are evaluated by different professionals (physicians and nurses) at each stage, may negatively affect the reliability of the questionnaire, particularly in the domains of satisfaction with care and expectations.

Nine versions of the instrument were applied, including pre- and postoperative modules, which have different items: two for mastectomy without reconstruction (pre and post), two for reconstruction short form (pre and post), two for reconstruction long form (pre and post), two for BCT (pre and post), and one for BCT associated with radiotherapy (post). The database developed at REDCap/HCB included 920 variables per patient, reflecting the complexity and comprehensiveness of the data collection process. For the analysis of convergent and discriminant validity, various quality of life instruments previously validated for Brazilian Portuguese were used, allowing comparisons between BREAST-Q domains and correlated scales.

Among the methodological strengths of the present study is the application of questionnaires in both pre- and postoperative periods, an approach often neglected or conducted with separate samples. Additionally, the study used updated and specific instruments for breast cancer, such as EORTC QLQ-BR42. In the postoperative period, three comparison instruments were applied: EORTC QLQ-C30 (general), EORTC QLQ-BR42 (breast cancer-specific), and EORTC QLQ-BRECON23 (specific to breast reconstruction), allowing for a thorough and accurate validation of BREAST-Q domains. Although, EORTC QLQ-BR42 was recently published and EORTC QLQ-BRECON23 was not compared to Breast-Q, high agreement was observed between the questionnaires, and this is the first study to show these results.

At this stage, internal structure analyses such as exploratory/confirmatory factor analysis and RASCH model were not performed due to the extensive length of the questionnaire, multiple methodologies used for validation, and limited number of patients observed in some groups. We opted to performing secondary analysis in other study, grouping surgical conditions, focusing on other validation instruments of Breast-Q, as no study evaluated factorial analysis, and we evaluated immediate postoperative period.

The results of this study are applicable to patients treated at a tertiary hospital within the Brazilian Unified Health System (SUS). Additional studies are needed to assess the generalizability of these findings to other healthcare models in Brazil, such as private practice and the supplementary health sector. However, this limitation does not compromise the validation of the instrument for Brazilian Portuguese, as approximately 70% of breast cancer cases under treatment in the country occur within the SUS.

Future perspectives of this research area include the implementation of the electronic version in clinical practice, along with the incorporation of additional assessment methods to evaluate surgical outcomes and monitor variations in quality of life throughout follow-up.

## 5 Conclusion

The findings of this study demonstrate that all breast cancer modules of the BREAST-Q are valid and reliable for assessing quality of life among Brazilian women who have undergone surgical treatment for breast cancer. The instrument exhibited adequate internal consistency and test–retest reliability, as well as strong convergent and discriminant validity, displaying strong correlation with other previously validated quality of life questionnaires. These results support the use of the BREAST-Q as a robust tool for evaluating patient-centered outcomes in clinical practice and research in the Brazilian context.

## Data Availability

The raw data supporting the conclusions of this article will be made available by the authors, without undue reservation.

## References

[1] Allemani C Matsuda T Di Carlo V Harewood R Matz M Niksic M . Global surveillance of trends in cancer survival 2000-14 (CONCORD-3): analysis of individual records for 37 513–025 patients diagnosed with one of 18 cancers from 322 population-based registries in 71 countries. Lancet. (2018) 391:1023–75. doi: 10.1016/S0140-6736(17)33326-3, PMID: 29395269 PMC5879496

[2] Cavalcante FP Millen EC Zerwes FP Novita GG . Progress in local treatment of breast cancer: A narrative review. Rev Bras Ginecol Obstet. (2020) 42:356–64. 10.1055/s-0040-1712125 PMC10418127 32604439

[3] Vieira R Bailao-Junior A de Oliveira-Junior I . Does breast oncoplastic surgery improve quality of life? Front Oncol. (2022) 12:1099125. 36713564 10.3389/fonc.2022.1099125 PMC9877289

[4] Pusic AL Chen CM Cano S Klassen A McCarthy C Collins ED . Measuring quality of life in cosmetic and reconstructive breast surgery: a systematic review of patient-reported outcomes instruments. Plast Reconstr Surg. (2007) 120:823–37. doi: 10.1097/01.prs.0000278162.82906.81, PMID: 17805107

[5] Chen CM Cano SJ Klassen AF King T McCarthy C Cordeiro PG . Measuring quality of life in oncologic breast surgery: a systematic review of patient-reported outcome measures. Breast J. (2010) 16:587–97. doi: 10.1111/j.1524-4741.2010.00983.x, PMID: 21070435

[6] Pusic AL Klassen AF Scott AM Klok JA Cordeiro PG Cano SJ . Development of a new patient-reported outcome measure for breast surgery: the BREAST-Q. Plast Reconstr Surg. (2009) 124:345–53. doi: 10.1097/PRS.0b013e3181aee807, PMID: 19644246

[7] Fuzesi S Cano SJ Klassen AF Atisha D Pusic AL . Validation of the electronic version of the BREAST-Q in the army of women study. Breast. (2017) 33:44–9. doi: 10.1016/j.breast.2017.02.015, PMID: 28279888 PMC5551502

[8] Klassen AF Dominici L Fuzesi S Cano SJ Atisha D Locklear T . Development and validation of the BREAST-Q breast-conserving therapy module. Ann Surg Oncol. (2020) 27:2238–47. doi: 10.1245/s10434-019-08195-w, PMID: 31965369 PMC8174155

[9] Tsangaris E Klassen AF Kaur MN Voineskos S Bordeleau L Zhong T . Development and psychometric validation of the BREAST-Q sensation module for women undergoing post-mastectomy breast reconstruction. Ann Surg Oncol. (2021) 28:7842–53. doi: 10.1245/s10434-021-10094-y, PMID: 33988795

[10] Cohen WA Mundy LR Ballard TN Klassen A Cano SJ Browne J . The BREAST-Q in surgical research: A review of the literature 2009-2015. J Plast Reconstr Aesthet Surg. (2016) 69:149–62. doi: 10.1016/j.bjps.2015.11.013, PMID: 26740288 PMC4995882

[11] Cano SJ Klassen AF Scott AM Pusic AL . A closer look at the BREAST-Q((c)). Clin Plast Surg. (2013) 40:287–96. doi: 10.1016/j.cps.2012.12.002, PMID: 23506769

[12] Saiga M Taira N Kimata Y Watanabe S Mukai Y Shimozuma K . Development of a Japanese version of the BREAST-Q and the traditional psychometric test of the mastectomy module for the assessment of HRQOL and patient satisfaction following breast surgery. Breast Cancer. (2017) 24:288–98. doi: 10.1007/s12282-016-0703-6, PMID: 27179527

[13] Shunck TS Veiga DF Cavagna FA Le GJr Neto MS Ferreira LM . Brazilian version of the Breast-Q((c)) -Breast-Conserving Therapy Module 2.0: Translation, cross-cultural adaptation, and reproducibility. Breast J. (2021) 27:72–4. doi: 10.1111/tbj.14064, PMID: 32989894

[14] Willert CB Gjorup CA Holmich LR . Danish translation and linguistic validation of the BREAST-Q. Dan Med J. (2020) 67:A08190445. 32351200

[15] Meireles R Tome G Pinheiro S Diogo C . BREAST-Q translation and linguistic validation to european portuguese. Acta Med Port. (2022) 35:823–9. doi: 10.20344/amp.17427, PMID: 35791701

[16] Weick L Grimby-Ekman A Lunde C Hansson E . Validation and reliability testing of the BREAST-Q expectations questionnaire in Swedish. J Plast Surg Handb Surg. (2023) 57:315–23. doi: 10.1080/2000656X.2022.2070180, PMID: 35533094

[17] Shunnmugam B Islam T Sinnadurai S Seng Hui C Mee Hong S Chinna K . Reliability and validity of the malay BREAST-Q in women undergoing breast cancer surgery in Malaysia. Asia Pac J Public Health. (2023) 35:129–35. doi: 10.1177/10105395231163345, PMID: 37171249

[18] Martinez-Perez JL Pascual-Dapena A Pardo Y Ferrer M Pont A Lopez MJ . Validation of the Spanish electronic version of the BREAST-Q questionnaire. Eur J Surg Oncol. (2023) 49:1417–22. doi: 10.1016/j.ejso.2023.04.011, PMID: 37179146

[19] Olasehinde O Lynch KA Goldman DA Agodirin O Okereke C Wuraola FO . Translation and psychometric assessment of the mastectomy module of the BREAST-Q questionnaire for use in Nigeria. J Patient Rep Outcomes. (2024) 8:17. doi: 10.1186/s41687-024-00692-1, PMID: 38334903 PMC10857998

[20] Homsy P Repo J Kuhlefelt C Lindford A Ihalainen H Kauhanen S . Finnish translation, validation, and reproducibility of BREAST-Q modules relevant to breast cancer treatment. Scand J Surg. (2025) 114:65–72. doi: 10.1177/14574969241277636, PMID: 39308151

[21] Cordanto-Nopoulos FR Sbalchiero JC Silva CHD Caiado-Neto BR Derchain S . Traduação do questionário BREAST-Q para a língua portuguesa e sua aplicação em mulheres com câncer de mama. Rev Bras Cir Plast. (2013) 28:79.

[22] Cammarota MC Campos AC Faria CADC Dos-Santos GC Barcelos LDP Mendonça FT . Quality of life and aesthetic results after mastectomy and mammary reconstruction. Rev Bras Cir Plást. (2019) 34:45–57. doi: 10.5935/2177-1235.2019RBCP0008-EN, PMID: 36351073

[23] Oliveira IGE . Breast-Q - breast reconstruciton expectations module (Pre operative). Tradução e adaptação cultural para o Brasil. São Paulo: Unidade Federal de São Paulo Rev Assoc Med Bras. (2022) 68:498–501. doi: 10.1590/1806-9282.20211095. PMID: 35649073

[24] Bjelic-Radisic V Cardoso F Weis J Pogoda K Arraras JI Greimel E . An international Phase IV field study - psychometric properties of the updated module on assessing quality of life of patients with breast cancer EORTC QLQ-BR42. Breast. (2025) 80:103890. doi: 10.1016/j.breast.2025.103890, PMID: 39947087 PMC11867226

[25] Winters ZE Afzal M Rutherford C Holzner B Rumpold G da Costa Vieira RA . International validation of the European Organisation for Research and Treatment of Cancer QLQ-BRECON23 quality-of-life questionnaire for women undergoing breast reconstruction. Br J Surg. (2018) 105:209–22. doi: 10.1002/bjs.10656, PMID: 29116657

[26] Stanton AL Krishnan L Collins CA . Form or function? Part 1. Subjective cosmetic and functional correlates of quality of life in women treated with breast-conserving surgical procedures and radiotherapy. Cancer. (2001) 91:2273–81. doi: 10.1002/1097-0142(20010615)91:12<2273::AID-CNCR1258>3.0.CO;2-1 11413515

[27] Vieira R Silva F Silva MES Silva JJD Sarri AJ Paiva CE . Translation and cultural adaptation of the Breast Cancer Treatment Outcome Scale (BCTOS) into Brazilian Portuguese. Rev Assoc Med Bras (1992). (2018) 64:627–34. doi: 10.1590/1806-9282.64.07.627, PMID: 30365665

[28] Santos TBL Pinto RMC . Tamanho de amostra para o teste-reteste na determinação do coeficiente de correlação interclasse. Universidade Federal de Uberlândia: Uberlândia (2018). Available online at: https://repositorio.ufu.br/bitstream/123456789/22300/1/TamanhoAmostraTesteReteste.pdf (Accessed February 17, 2026).

[29] Jagsi R Li Y Morrow M Janz N Alderman A Graff J . Patient-reported quality of life and satisfaction with cosmetic outcomes after breast conservation and mastectomy with and without reconstruction: results of a survey of breast cancer survivors. Ann Surg. (2015) 261:1198–206. doi: 10.1097/SLA.0000000000000908, PMID: 25654742 PMC4512928

[30] Kim MK Kim T Moon HG Jin US Kim K Kim J . Effect of cosmetic outcome on quality of life after breast cancer surgery. Eur J Surg Oncol. (2015) 41:426–32. doi: 10.1016/j.ejso.2014.12.002, PMID: 25578249

[31] Stolpner I Heil J Feisst M Karsten MM Weber WP Blohmer JU . Clinical validation of the BREAST-Q breast-conserving therapy module. Ann Surg Oncol. (2019) 26:2759–67. doi: 10.1245/s10434-019-07456-y, PMID: 31115853

[32] Gu J Groot G Boden C Busch A Holtslander L Lim H . Review of factors influencing women’s choice of mastectomy versus breast conserving therapy in early stage breast cancer: A systematic review. Clin Breast Cancer. (2018) 18:e539–e54. doi: 10.1016/j.clbc.2017.12.013, PMID: 29396079

[33] Gupta D Rodeghier M Lis CG . Patient satisfaction with service quality as a predictor of survival outcomes in breast cancer. Support Care Cancer. (2014) 22:129–34. doi: 10.1007/s00520-013-1956-7, PMID: 24013568

[34] Stolpner I Hennigs A . Response to: Comments on the Clinical Validation of the BREAST-Q Breast-Conserving Therapy Module, by Hernanz et al. Ann Surg Oncol. (2019) 26:857–8. doi: 10.1245/s10434-019-07756-3, PMID: 31463698

[35] Hernanz F Jimeno J Paz L Munoz P . Comments on the clinical validation of the BREAST-Q breast-conserving therapy module. Ann Surg Oncol. (2019) 26:855–6. doi: 10.1245/s10434-019-07754-5, PMID: 31440930

[36] Vohra LM Javed SM Jabeen D Abidi SS Tahseen MU . Quality of life of breast cancer survivors: a comparison of breast conserving surgery versus total mastectomy with and without immediate reconstruction: a prospective cohort study. Ann Med Surg (Lond). (2023) 85:1513–7. doi: 10.1097/MS9.0000000000000607, PMID: 37228946 PMC10205257

[37] Blok YL Verduijn PS Corion LUM Visser JM van der Pol CC van der Hage JA . An analysis of complication rates and the influence on patient satisfaction and cosmetic outcomes following oncoplastic breast surgery. J Plast Reconstr Aesthet Surg. (2022) 75:4152–9. doi: 10.1016/j.bjps.2022.06.088, PMID: 36171174

[38] Flitcroft K Brennan M Spillane A . Women’s expectations of breast reconstruction following mastectomy for breast cancer: a systematic review. Support Care Cancer. (2017) 25:2631–61. doi: 10.1007/s00520-017-3712-x, PMID: 28474240

