# Supplementary material for: Brazilian Portuguese validation of the breast cancer-related modules of the BREAST-Q
Source: Front Oncol. 2026 Apr 6;16:1674229. doi: 10.3389/fonc.2026.1674229 (PMC13093880; doi:10.3389/fonc.2026.1674229)
Supplement: Supplementary file 1 [file DataSheet1.pdf]

## SUPPLEMENTARY TABLES


| | | |
|---|---|---|
| ST 1 | Sample size calculation for $\alpha$ values ranging from 0.70 to 0.75 …………… | 2 |
| ST 2 | Data collection periods……………………………………………………... | 3 |
| ST 3 | Comparison between EORTC and the BREAST-Q………………………… | 4 |
| ST 4 | Comparison between the new EORTC* and BREAST-Q……..…………… | 6 |
| ST 5 | Response rate of preoperative BREAST-Q modules……………………… | 7 |
| ST 6 | Response rate of postoperative BREAST-Q modules……………………… | 9 |
| ST 7 | Internal consistency of preoperative and postoperative BREAST-Q modules ……………………………………..…………………………... | 11 |
| ST 8 | Internal consistency of the breast-conserving therapy (BCT) module of the BREAST-Q ……………………………..……………………………. | 12 |
| ST9 | Internal consistency of the breast conserving therapy (BCT) with radiotherapy module of the BREAST-Q …………………..………………… | 13 |
| ST 10 | Internal consistency of the reconstruction (long form) module of the BREAST-Q ……………………………………...…………………… | 14 |
| ST 11 | Internal consistency of the reconstruction (short form) module of the BREAST-Q ……………………………………...…………………… | 15 |
| ST 12 | Internal consistency of the mastectomy without reconstruction module of the BREAST-Q………………………………..………………………… | 16 |
| ST 13 | Test–retest reliability of the BREAST-Q………………………………… | 17 |
| ST 14 | Internal consistency of the expectations module…………………………… | 19 |
| ST 15 | Mean scores of questionnaires for the preoperative period……..………… | 22 |
| ST 16 | Mean scores of questionnaires for the postoperative period…..…………… | 24 |

**TABLE S1.** Sample size calculation for *α* values ranging from 0.70 to 0.75.

| Factor | Reconstruction | Breast-conserving therapy | Mastectomy | All |
|---|---|---|---|---|
| Satisfaction with breasts | 12 | 12 | 66 | - |
| Adverse effects of radiation | | 54 ($\alpha$ = 0.70) | - | - |
| Satisfaction with outcome | 25 | - | - | - |
| Psychosocial well-being | 12 | 13 | 13 | - |
| Sexual well-being | 16 | 17 | 16 | - |
| Physical well-being | 17 | 25 | 16 | - |
| Satisfaction with information | 13 | 16 | - | 11 |
| Satisfaction with surgeon | - | - | - | 12 |
| Satisfaction with medical team | - | - | - | 12 |
| Minimum/preoperative module | 25* | 25 | 66 | 12 |
| Radiotherapy (postoperative) | - | 54 | - | - |
| Total number of patients | 50 | 79 | 66 | - |

*Short and long forms.

**TABLE S2.** Data collection periods.

| Questionnaire | Period |
|---|---|
| **Preoperative** | |
| Sociodemographic data | Day 1 preoperative |
| Specific preoperative questionnaire | Day 1 preoperative |
| EORTC QLQ-C30, BR45 | Day 1 preoperative |
| Preoperative test/retest | Days 7–14 |
| | |
| **Postoperative** | |
| Specific postoperative questionnaire | Day 7 postoperative |
| EORTC QLQ-C30, BR45 | Day 7 postoperative |
| BRECON23 | Day 7 postoperative |
| Postoperative test/retest | Days 7–14 |
| Surgical complication form | Days 7–37 postoperative |
| | |
| **Radiotherapy (breast-conserving therapy)** | |
| Sociodemographic data | Day 1 |
| Specific postoperative questionnaire | Day 1 |
| EORTC QLQ-C30, BR45, BCTOS | Day 1 |
| Preoperative test/retest | Days 7–14 |

**TABLE S3.** Comparison between EORTC and the BREAST-Q.

| Questionnaire | | | BREAST-Q well-being | | | BREAST-Q satisfaction | | |
|---|---|---|---|---|---|---|---|---|
| | | | Physical | Psychosocial | Sexual | Breast | Outcome | Care |
| **QLQ-C30** | Global health | | x | | | | | |
| | Function scales | Physical functioning | x | | | | | |
| | | Role functioning | x | | | | | |
| | | Emotional functioning | | x | | | x | |
| | | Cognitive functioning | | | | | | |
| | | Social functioning | | x | | | | |
| | Symptom scales | Fatigue | | | | | | |
| | | Nausea and vomiting | | | | | | |
| | | Pain | | | | | | |
| | | Dyspnea | | | | | | |
| | | Insomnia | | | | | | |
| | | Appetite Loss | | | | | | |
| | | Constipation | | | | | | |
| | | Diarrhea | | | | | | |
| | | Financial difficulties | | | | | | |
| **QLQ-BR23** | Function scales | Body image | x | | | x | x | |
| | | Sexual functioning | | | x | | | |
| | | Sexual enjoyment | | | x | | | |
| | Symptom scales | Systemic therapy side effects | x | | | | | |
| | | Breast symptoms | | | | x | | |
| | | Arm symptoms | x | | | | | |
| | | Upset by hair loss | | | | | | |

**TABLE S4.** Comparison between the new EORTC* and BREAST-Q.

| **Questionnaire** | | | **BREAST-Q well-being** | | | **BREAST-Q satisfaction** | | |
|---|---|---|---|---|---|---|---|---|
| | | | **Physical** | **Psychosocial** | **Sexual** | **Breast** | **Outcome** | **Care** |
| **QLQ-BR23** | Function scales | Body image | x | | | x | x | |
| | | Sexual functioning | | | x | | | |
| | | Sexual enjoyment | | | x | | | |
| | Symptom scales | Systemic therapy side effects | x | | | | | |
| | | Breast symptoms | | | | x | | |
| | | Arm symptoms | x | | | | | |
| | | Upset by hair loss | | | | | | |
| **QLQ-BR45 (update)** | Function scales | Breast satisfaction | | | | x | | |
| | Target therapy | Endocrine therapy symptoms | | | | | | |
| | | Endocrine sexual symptoms | | | x | | | |
| | | Skin mucositis | | | | | | |
| **BRECON23** | Scales | Surgical side-effects | | | | | | |
| | | Sexuality | | | x | | | |
| | | Satisfaction with breast cosmetic | | | | x | | |
| | | Satisfaction with nipple cosmetic | | | | x | | |
| | | Satisfaction with surgery | | | | x | | |
| | | Satisfaction with donor scars | | | | x | | |

| | | | | | | | | |
|---|---|---|---|---|---|---|---|---|
| | Simple items | Finding a well-fitting bra | | | | | x | |
| | | Satisfaction with donor scars | | | | | x | |
| | | Loss of nipple | | | | | x | |
| | | Preserve/reconstruct nipple | | | | | x | |
| **BCTOS** | | Functional | x | | | | | |
| | | Cosmetic | | | | | x | |
| | | Breast pain | | | | | x | |
| | | Edema | x | | | | | |

*EORTC-QLQ items are found in EORTC-QLQ BR45.

**TABLE S5.** Response rate of preoperative BREAST-Q modules.

| Domain | No. of items | Response rate (%) | | | | |
|---|---|---|---|---|---|---|
| | | Mastectomy | Reconstruction (short form) | Reconstruction (long form) | BCT | BCT with radiotherapy |
| Psychosocial well-being | 10 | a100% | a100% | a100% | a100% | - |
| | | b100% | b100% | b100% | b100% | - |
| | | c100% | c100% | c96% | c100% | - |
| | | d100% | d100% | d100% | d100% | - |
| | | e100% | e100% | e100% | e100% | - |
| | | f96.96% | f100% | f100% | f100% | - |
| | | g100% | g100% | g100% | g100% | - |
| | | h100% | h100% | h100% | h100% | - |
| | | i100% | i100% | i100% | i100% | - |
| | | j100% | j100% | j96% | j100% | - |
| Physical well-being: chest | 10 | a100% | a 100% | a100% | a100% | - |
| | | b100% | b100% | b100% | b100% | - |
| | | c100% | c100% | c100% | c100% | - |
| | | d100% | d100% | d100% | d100% | - |
| | | e100% | e100% | e100% | e100% | - |
| | | f100% | f100% | f100% | f100% | - |
| | | g100% | g100% | g100% | g100% | - |
| | | h100% | h100% | h92% | h100% | - |
| | | i100% | i100% | i100% | i100% | - |
| | | j100% | j100% | j96% | j100% | - |
| Sexual well-being | 6 | a93.93% | a96% | a92% | a96.15% | - |
| | | b81.81% | b88% | b88% | b73.07% | - |
| | | c87.87% | c88% | c88% | c96.15% | - |

| | | | | | | |
|---|---|---|---|---|---|---|
| | | d87.87% | d88% | d88% | d80.76% | - |
| | | e89.39% | e88% | e92% | e96.15% | - |
| | | F90.90% | f88% | f92% | f96.15% | - |
| Satisfaction with breasts | 4 | a96.96% | a100% | a100% | a100% | - |
| | | b93.93% | b100% | b100% | b100% | - |
| | | c95.45% | c92% | c100% | c92.30% | - |
| | | d95.45% | d96% | d100% | d100% | - |

BCT= breast-conserving therapy.

**TABLE S6.** Response rate of postoperative BREAST-Q modules.

| Domain | No. of items | Response rate (%) | | | | |
|---|---|---|---|---|---|---|
| | | Mastectomy | Reconstruction (short form) | Reconstruction (long form) | BCT | BCT with radiotherapy |
| Psychosocial well-being | 10 | a100% | a100% | a100% | a100% | a100% |
| | | b100% | b100% | b100% | b100% | b100% |
| | | c100% | c100% | c96% | c100% | c100% |
| | | d100% | d100% | d100% | d100% | d100% |
| | | e100% | e100% | e100% | e100% | e100% |
| | | f100% | f100% | f100% | f100% | f100% |
| | | g100% | g100% | g100% | g100% | g98.14% |
| | | h100% | h100% | h100% | h100% | h100% |
| | | i100% | i100% | i100% | i100% | i100% |
| | | j100% | j100% | 100% | j100% | j96.29% |
| Physical well-being: chest | 10 | a 100% | a96% | a100% | a100% | a98% |
| | | b 100% | b96% | b96% | b100% | b100% |
| | | c 100% | c96% | c100% | c100% | c100% |
| | | d 100% | d100% | d100% | d100% | d100% |
| | | e 100% | e96% | e96% | e100% | e98% |
| | | f 98.48% | f100% | f100% | f100% | f100% |
| | | g 100% | g96% | g100% | g100% | g100% |
| | | h 100% | h100% | h100% | h100% | - |
| | | i 98.48% | i100% | i100% | i100% | - |
| | | j 100% | j100% | j100% | j100% | - |
| Sexual well-being | 6 | a93.93% | a96% | a92% | a100% | a96.29% |
| | | b72.70% | b72% | b92% | b80.76% | b92.59% |
| | | c78.70% | c72% | c92% | c84.61% | c92.59% |

| | | | | | | |
|---|---|---|---|---|---|---|
| | | d81.80%<br>e86.30%<br>F86.30% | d72%<br>e72%<br>f76% | d92%<br>e88%<br>f88% | d84.61%<br>e92.30%<br>f92.30% | d92.59%<br>e96.29%<br>f96.29% |
| Satisfaction with breast | 15 | a98.40%<br>b92.42%<br>c92.42%<br>d96.96%<br>e-<br>f-<br>g-<br>h-<br>i-<br>j-<br>k-<br>l-<br>m-<br>n-<br>o- | a84%<br>b88%<br>c88%<br>d88%<br>e84%<br>f84%<br>g80%<br>h84%<br>i88%<br>j88%<br>k88%<br>l84%<br>m88%<br>n88%<br>o88% | a100%<br>b100%<br>c100%<br>d100%<br>e96%<br>f100%<br>g100%<br>h100%<br>i100%<br>j100%<br>k100%<br>l100%<br>m100%<br>n100%<br>o100% | a100%<br>b100%<br>c100%<br>d100%<br>e100%<br>f100%<br>g100%<br>h100%<br>i100%<br>j100%<br>k100%<br>l-<br>m-<br>n-<br>o- | a98.14%<br>b98.14%<br>c100%<br>d100%<br>e100%<br>f98.14%<br>g100%<br>h100%<br>i100%<br>j98.14%<br>k100%<br>l-<br>m-<br>n-<br>o- |

BCT= breast-conserving therapy.

**TABLE S7.** Internal consistency of preoperative and postoperative BREAST-Q modules.

| Module | Cronbach's α test | Cronbach's α retest | *p*-value |
|---|---|---|---|
| **Preoperative** | | | |
| Mastectomy | 0.81 | 0.89 | <0.001 |
| Reconstruction (short form) | 0.80 | 0.80 | <0.001 |
| Reconstruction (long form) | 0.82 | 0.91 | <0.001 |
| BCT | 0.89 | O.91 | <0.001 |
| **Postoperative** | | | |
| Mastectomy | 0.93 | 0.92 | <0.001 |
| Reconstruction (short form) | 0.96 | 0.97 | <0.001 |
| Reconstruction (long form) | 0.81 | 0.85 | <0.001 |
| BCT | 0.87 | 0.94 | <0.001 |
| BCT with radiotherapy | 0.89 | 0.95 | <0.001 |

BCT= breast-conserving therapy.

**TABLE S8** Internal consistency of the breast-conserving therapy (BCT) module of the BREAST-Q.

| Domain | Preoperative | | | | Postoperative | | | |
|---|---|---|---|---|---|---|---|---|
| | **No. of items** | ***n*** | **Cronbach's *α*** | ***p*-value** | **No. of items** | ***n*** | **Cronbach's *α*** | ***p*-value** |
| Psychosocial well-being | 10 | 25 | 0.85* | <0.001 | 10 | 25 | 0.92* | <0.001 |
| Physical well-being: chest | 10 | 25 | 081* | <0.001 | 10 | 25 | 0.86 | <0.001 |
| Sexual well-being | 6 | 25 | 0.92* | <0.001 | 6 | 25 | 0.89 | <0.001 |
| Satisfaction with breasts | 4 | 25 | 0.77 | <0.001 | 15 | 25 | 0.93 | <0.001 |
| Satisfaction with information | - | - | - | | 12 | 25 | 0.77 | <0.001 |
| Satisfaction with surgeon | - | - | - | | 12 | 25 | 0.52 | |
| Satisfaction with medical team | - | - | - | | 7 | 25 | 0.52 | <0.001 |
| Satisfaction with office staff | - | - | - | | 7 | 25 | 0.12 | <0.001 |

**TABLE S9** Internal consistency of the breast conserving therapy (BCT) with radiotherapy module of the BREAST-Q.

| **Domain** | **Postoperative** | | | |
|---|---|---|---|---|
| | **No. of items** | ***n*** | **Cronbach's *α*** | ***p*-value** |
| Psychosocial well-being | 10 | 51 | 0.94 | <0.001 |
| Physical well-being: chest | 10 | 52 | 0.85 | <0.001 |
| Sexual well-being | 6 | 50 | 0.89 | <0.001 |
| Adverse effects of radiation | 6 | 52 | 0.81 | <0.001 |
| Satisfaction with breasts | 15 | 52 | 0.92 | <0.001 |
| Satisfaction with information: breast surgeon | 12 | 51 | 0.84 | <0.001 |
| Satisfaction with information: radiation oncologist | 11 | 51 | 0.95 | <0.001 |

**TABLE S10** Internal consistency of the reconstruction (long form) module of the BREAST-Q.

| Domain | Preoperative | | | | Postoperative | | | |
|---|---|---|---|---|---|---|---|---|
| | **No. of items** | ***n*** | **Cronbach's *α*** | ***p*-value** | **No. of items** | ***n*** | **Cronbach's *α*** | ***p*-value** |
| Psychosocial well-being | 10 | 23 | 0.91 | <0.001 | 10 | 25 | 0.92 | <0.001 |
| Physical well-being: chest | 10 | 22 | 0.62 | <0.001 | 10 | 23 | 0.53 | <0.001 |
| Sexual well-being | 6 | 22 | 0.86 | <0.001 | 6 | 22 | 0.90 | <0.001 |
| Satisfaction with breasts | 4 | 25 | 0.84 | <0.001 | 15 | 24 | 0.96 | <0.001 |
| Satisfaction with information | - | - | - | | 12 | 23 | 0.76 | <0.001 |
| Satisfaction with surgeon | - | - | - | | 12 | 23 | 0.51 | |
| Satisfaction with medical team | - | - | - | | 7 | 25 | 0.54 | <0.001 |
| Satisfaction with office staff | - | - | - | | 7 | 24 | 0.53 | <0.001 |
| Expectations for medical team | 5 | 25 | 0.35 | <0.001 | - | - | - | - |
| Expectations for pain | 6 | 25 | 0.82 | <0.001 | - | - | - | - |
| Expectations for coping | 5 | 25 | 0.83 | <0.001 | - | - | - | - |
| Expectations for appearance | 5 | 25 | 0.62 | <0.001 | - | - | - | - |
| Expectations for implants | 5 | 24 | 0.44 | <0.001 | - | - | - | - |

**TABLE S11** Internal consistency of the reconstruction (short form) module of the BREAST-Q.

| **Domain** | **Preoperative** | | | | **Postoperative** | | | |
|---|---|---|---|---|---|---|---|---|
| | **No. of items** | ***n*** | **Cronbach's *α*** | ***p*-value** | **No. of items** | ***n*** | **Cronbach's *α*** | ***p*-value** |
| Psychosocial well-being | 10 | 24 | 0.90 | <0.001 | 10 | 24 | 0.96 | <0.001 |
| Physical well-being: chest | 10 | 24 | 0.85 | <0.001 | 10 | 23 | 0.77 | <0.001 |
| Sexual well-being | 6 | 20 | 0.93 | <0.001 | 6 | 16 | 0.94 | <0.001 |
| Satisfaction with breasts | 4 | 23 | 0.81 | <0.001 | 15 | 16 | 0.96 | <0.001 |
| Satisfaction with information | - | - | - | | 15 | 23 | 0.87 | <0.001 |
| Satisfaction with surgeon | - | - | - | | 12 | | 0.89 | <0.001 |
| Satisfaction with medical team | - | - | - | | 7 | 24 | 0.84 | <0.001 |
| Satisfaction with office staff | - | - | - | | 7 | 22 | 0.92 | <0.001 |
| Expectations for pain | 6 | 25 | 0.86 | | - | - | - | - |
| Expectations for appearance | 5 | 25 | 0.81 | | - | - | - | - |

**TABLE S12** Internal consistency of the mastectomy without reconstruction module of the BREAST-Q.

| Domain | Preoperative | | | | Postoperative | | | |
|---|---|---|---|---|---|---|---|---|
| | **No. of items** | ***n*** | **Cronbach's *α*** | ***p*-value** | **No. of items** | ***n*** | **Cronbach's *α*** | ***p*-value** |
| Psychosocial well-being | 10 | 62 | 0.93 | <0.001 | 10 | 66 | 0.97 | <0.001 |
| Physical well-being: chest | 10 | 66 | 087 | <0.001 | 10 | 65 | 0.85 | <0.001 |
| Sexual well-being | 6 | 51 | 0.92 | <0.001 | 6 | 48 | 0.89 | <0.001 |
| Satisfaction with breasts | 4 | 60 | 0.67 | <0.001 | 4 | 57 | 0.94 | <0.001 |
| Satisfaction with surgeon | - | - | - | | 12 | 65 | 0.88 | <0.001 |
| Satisfaction with medical team | - | - | - | | 7 | 64 | 0.87 | <0.001 |
| Satisfaction with office staff | - | - | - | | 7 | 65 | 0.88 | <0.001 |

**TABLE S13** Test–retest reliability of the BREAST-Q.

| Domain | Preoperative | | | | Postoperative | | | |
|---|---|---|---|---|---|---|---|---|
| | No. of items | *n* | ICC | 95% CI | No. of items | *n* | ICC | 95% CI |
| **Reconstruction (short form)** | | | | | | | | |
| Psychosocial well-being | 10 | 25 | 0.86 | 0.69–0.94 | 10 | 25 | 0.88 | 0.74–0.95 |
| Physical well-being: chest | 10 | 25 | 0.93 | 0.85–0.7 | 10 | 25 | 0.97 | 0.93–0.98 |
| Sexual well-being | 6 | 20 | 0.87 | 0.69–0.95 | 6 | 18 | 0.84 | 0.60–0.94 |
| Satisfaction with breasts | 4 | 24 | 0.79 | 0.51–0.91 | | 19 | 0.97 | 0.92–0.99 |
| **Reconstruction (long form)** | | | | | | | | |
| Psychosocial well-being | 10 | 24 | 0.84 | 0.64–0.93 | 10 | 25 | 0.90 | 0.79–0.96 |
| Physical well-being: chest | 10 | 25 | 0.92 | 0.83–0.96 | 10 | 25 | 0.72 | 0.35–0.87 |
| Sexual well-being | 6 | 22 | 0.90 | 0.77–0.96 | 6 | 22 | 0.87 | 0.69–0.94 |
| Satisfaction with breasts | 4 | 25 | 0.79 | 0.53–0.90 | 15 | 25 | 0.72 | 0.36–0.88 |
| **Mastectomy** | | | | | | | | |
| Psychosocial well-being | 10 | 66 | 0.89 | 0.82–0.93 | 10 | 66 | 0.94 | 0.90–0.96 |
| Physical well-being: chest | 10 | 66 | 0.95 | 0.91–0.97 | 10 | 66 | 0.79 | 0.66–0.87 |
| Sexual well-being | 6 | 54 | 0.97 | 0.95–0.98 | 6 | 51 | 0.93 | 0.89–0.96 |
| Satisfaction with breasts | 4 | 64 | 0.85 | 0.75–0.90 | 15 | 64 | 0.93 | 0.89–0.96 |
| **Breast-conserving therapy** | | | | | | | | |
| Psychosocial well-being | 10 | 25 | 0.87 | 0.71–0.94 | 10 | 25 | 0.85 | 0.66–0.93 |
| Physical well-being: chest | 10 | 25 | 0.76 | 0.46–0.89 | 10 | 25 | 0.74 | 0.41–0.88 |
| Sexual well-being | 6 | 24 | 0.72 | 0.70–0.79 | 6 | 22 | 0.94 | 0.85–0.98 |
| Satisfaction with breasts | 4 | 25 | 0.79 | 0.40–0.87 | 15 | 25 | 0.66 | 0.12–0.87 |
| **Breast-conserving therapy with radiotherapy** | | | | | | | | |
| Psychosocial well-being | - | - | - | – | 10 | 54 | 0.91 | 0.86–0.95 |
| Physical well-being: chest | - | - | - | – | 10 | 54 | 0.85 | 0.74–0.91 |
| Sexual well-being | - | - | - | – | 6 | 46 | 0.95 | 0.92–0.97 |

| | | | | | | | | |
|---|---|---|---|---|---|---|---|---|
| Satisfaction with breasts | - | - | - | – | 15 | 54 | 0.90 | 0.83–0.94 |

$p < 0.001$. ICC, intraclass correlation coefficient; CI, confidence interval.

**TABLE S14.** Internal consistency of the expectations module.

| **Item** | **Short form** | | | **Long form** | | |
|---|---|---|---|---|---|---|
| | ***n*** | **ICC** | **95% CI** | ***n*** | **ICC** | **95% CI** |
| How much information do you expect you will want before your upcoming breast reconstruction surgery? | - | - | - | 25 | 0.92 | 0.84–0.97 |
| How involved do you expect to be in making decisions about your upcoming breast reconstruction surgery? | - | - | - | 25 | 0.96 | 0.91–0.98 |
| What kind of support do you expect from the medical staff during the breast reconstruction process? (Expectations regarding the medical team) | | | | 25 | 0.72 | 0.36–0.88 |
| Imagine yourself in the first week after your breast reconstruction surgery. What do you expect the pain to be like? (Pain expectation) | 18 | 0.88 | 0.70–0.95 | 19 | 0.86 | 0.64–0.95 |
| Imagine yourself during the first year after breast reconstruction. How do you think you will cope with the process of reconstruction? (Coping expectation) | | | | 21 | 0.73 | 0.34–0.89 |
| Imagine yourself 1 year after your breast reconstruction. What do you expect to look like when you are in clothing? (Appearance expectation) | 18 | 0.84 | 0.58–0.94 | 21 | 0.23 | 0.78–0.68 |
| Imagine yourself 1 year after your breast reconstruction surgery. What do you expect your new breast(s) will feel like when you touch them? (Implant expectation) | | | | 22 | 0.80 | 0.50–0.92 |
| How likely do you think it is that you might experience a complication after your upcoming breast reconstruction surgery? | - | - | - | 25 | 0.94 | 0.87–0.97 |
| Imagine yourself 1 year after your breast reconstruction surgery. What do you expect your new breast(s) to look like when you are unclothed? | - | - | - | 24 | 0.98 | 0.96–0.99 |
| Imagine yourself 1 year after your breast reconstruction surgery. How similar (symmetric) do you expect your breasts to look when unclothed? | - | - | - | 24 | 0.98 | 0.96–0.99 |

| | | | | | | |
|---|---|---|---|---|---|---|
| Imagine yourself 1 year after your breast reconstruction surgery. How similar (symmetric) do you expect your breasts to look when unclothed? | 24 | 0.54 | <0.001 | 21 | 0.92 | <0.001 |
| Imagine yourself 1 year after your breast reconstruction surgery. How noticeable do you expect the scars on your breast(s) to be? | - | - | - | 24 | 0.92 | 0.82–0.96 |
| Imagine yourself 1 year after your breast reconstruction surgery. How much sensation (feeling) do you expect to have in your new breast(s)? | 24 | 0.80 | <0.001 | 24 | 0.91 | 0.80–0.96 |
| Imagine yourself 1 year after your breast reconstruction surgery. What size do you expect your breast(s) to be? | - | - | - | 24 | 0.56 | 0.01–0.81 |
| Imagine yourself 1 year after your breast reconstruction surgery. What do you expect the shape of your new breast(s) to look like? | - | - | - | 24 | 0.60 | 0.07–0.83 |
| Imagine yourself 1 year after your breast reconstruction surgery. How natural do you expect your breast(s) will look? | - | - | - | 24 | 0.88 | 0.72–0.95 |
| Imagine yourself 1 year after your breast reconstruction surgery. How much do you expect your new breast(s) will move? | - | - | - | 24 | 0.78 | 0.50–0.90 |
| Imagine yourself 1 year after your breast reconstruction surgery. How do you expect the sides of your chest (i.e. the area beside your breast under your arm) will look? | - | - | - | 24 | 0.85 | 0.66–0.94 |
| Imagine yourself 1 year after your breast reconstruction surgery. How much sensation (feeling) do you expect to have along the sides of your chest (i.e. the area beside your breast under your arm)? | - | - | - | 24 | 0.70 | 0.31–0.87 |
| Imagine yourself 1 year after your breast reconstruction surgery. How aware do you think you will be of your breast implant(s)? | - | - | - | 24 | 0.79 | 0.52–0.91 |
| Imagine yourself 1 year after your breast reconstruction surgery. What do you expect your nipple reconstruction will look like? | | | | 14 | 0.63 | 0.19–0.88 |
| Imagine yourself 1 year after your breast reconstruction surgery. How much sensation (feeling) do you expect to have in your nipple(s? | | | | 14 | 0.88 | 0.64–0.96 |

| | | | | | | |
|---|---|---|---|---|---|---|
| Imagine yourself 10 years after your breast reconstruction surgery. How similar (symmetric) do you expect your breasts to look? | - | - | - | 24 | 0.72 | 0.35–0.88 |
| Imagine yourself 10 years after your breast reconstruction surgery. Do you think you will need more reconstructive surgery on your breast(s)? | - | - | - | 24 | 0.81 | 0.57–0.92 |
| Imagine yourself 10 years after your breast reconstruction surgery. How similar (symmetric) do you expect your breasts to look? | 24 | 0.54 | 0.001 | - | - | |

ICC, intraclass correlation coefficient; CI, confidence interval; $p < 0.001$.

**TABLE S15.** Mean scores of questionnaires for the preoperative period.

| Questionnaire/item | Mastectomy | Reconstruction (short form) | Reconstruction (long form) | BCT | BCT with radiotherapy |
|---|---|---|---|---|---|
| **EORTC QLQ-C30** | | | | | |
| Physical functioning | 82.80 | 91.73 | 92.05 | 84.62 | - |
| Role functioning | 81.82 | 92.00 | 93.59 | 80.13 | - |
| Emotional functioning | 69.19 | 59.00 | 77.56 | 79.81 | - |
| Cognitive functioning | 82.07 | 78.67 | 84.62 | 67.95 | - |
| Social functioning | 86.11 | 87.33 | 91.67 | 91.03 | - |
| Fatigue | 47.98 | 17.33 | 04.49 | 24.36 | - |
| Nausea and vomiting | 21.21 | 14.67 | 05.98 | 10.68 | - |
| Pain | 02.02 | 04.67 | 02.56 | 05.77 | - |
| Dyspnea | 05.05 | 00.00 | 06.41 | 05.13 | - |
| Insomnia | 12.82 | 22.67 | 08.97 | 33.33 | - |
| Appetite loss | 04.55 | 05.33 | 01.28 | 19.23 | - |
| Constipation | 13.85 | 18.67 | 16.67 | 14.10 | - |
| Diarrhea | 02.02 | 10.67 | 02.67 | 01.28 | - |
| Financial difficulties | 15.38 | 22.67 | 01.28 | 23.08 | - |
| Quality of life | 73.48 | 78.00 | 76.92 | 74.68 | - |
| **EORTC QLQ-BR42** | | | | | |
| Body image | 85.23 | 84.89 | 85.58 | 85.58 | - |
| Future perspective | 23.23 | 37.33 | 25.64 | 44.00 | - |
| Sexual functioning | 33.08 | 54.00 | 53.21 | 25.68 | - |
| Sexual enjoyment | 43.10 | 66.67 | 68.00 | 40.74 | - |
| Systemic therapy side effects | 17.54 | 18.57 | 18.37 | 18.43 | - |
| Endocrine symptoms | 56.90 | 33.33 | 32.00 | 59.26 | - |
| Hand/Feet symptoms/ Neuropathy | 15.53 | 13.67 | 14.32 | 21.15 | - |

| | | | | | |
|---|---|---|---|---|---|
| Musculoskeletal symptoms | 06.82 | 10.67 | 06.09 | 14.10 | - |
| Weight gain | 15.65 | 22.67 | 12.00 | 17.95 | - |
| Vaginal symptoms | 10.00 | 20.00 | 11.97 | 17.33 | - |
| Breast symptoms | 18.31 | 14.93 | 08.65 | 09.62 | - |
| Arm symptoms | 14.48 | 13.78 | 06.84 | 21.79 | - |
| **BREAST-Q** | | | | | |
| Physical well-being: chest | 78.18 | 79.96 | 92.72 | 82.54 | - |
| Psychosocial well-being | 69.24 | 70.48 | 71.20 | 76.35 | - |
| Sexual well-being | 59.18 | 65.29 | 65.14 | 60.63 | - |
| Satisfaction breast | 69.17 | 79.38 | 69.76 | 67.69 | - |

BCT= breast-conserving therapy.

**TABLE S16.** Mean scores of questionnaires for the postoperative period.

| Questionnaire/item | Mastectomy | Reconstruction (short form) | Reconstruction (long form) | BCT | BCT with radiotherapy |
|---|---|---|---|---|---|
| **EORTC QLQ-C30** | | | | | |
| Physical functioning | 70.58 | 82.60 | 81.87 | 76.92 | 84.57 |
| Role functioning | 49.75 | 56.67 | 61.33 | 63.46 | 81.48 |
| Emotional functioning | 72.26 | 64.33 | 78.00 | 86.22 | 80.30 |
| Cognitive functioning | 85.61 | 86.67 | 94.67 | 82.69 | 82.10 |
| Social functioning | 75.51 | 81.33 | 97.33 | 82.69 | 93.52 |
| Fatigue | 47.98 | 44.00 | 36.67 | 24.36 | 20.99 |
| Nausea and vomiting | 21.21 | 19.11 | 13.78 | 18.38 | 13.37 |
| Pain | 02.02 | 02.00 | 2.00 | 4.49 | 3.09 |
| Dyspnea | 05.05 | 05.33 | 0.00 | 2.56 | 6.29 |
| Insomnia | 12.82 | 13.33 | 8.00 | 34.62 | 22.84 |
| Appetite Loss | 04.55 | 04.00 | 4.00 | 6.41 | 5.56 |
| Constipation | 13.85 | 20.00 | 16.00 | 21.79 | 15.72 |
| Diarrhea | 2.02 | 6.67 | 2.67 | 1.28 | 0.63 |
| Financial difficulties | 15.38 | 21.33 | 2.67 | 24.00 | 22.22 |
| Quality of life | 59.72 | 77.33 | 71.67 | 79.17 | 82.72 |
| **EORTC QLQ-BR42** | | | | | |
| Body image | 42.51 | 76.67 | 82.67 | 82.69 | 82.25 |
| Future perspective | 14.14 | 30.56 | 33.33 | 44.87 | 37.04 |
| Sexual functioning | 27.96 | 50.69 | 55.33 | 31.16 | 34.64 |
| Sexual enjoyment | 41.83 | 65.00 | 71.21 | 43.33 | 43.54 |
| Systemic therapy side effects | 15.21 | 12.24 | 7.93 | 12.34 | 10.82 |
| Endocrine symptoms | 13.38 | 16.27 | 10.13 | 15.38 | 19.60 |
| Hand/Feet symptoms/Neuropathy | 13.26 | 9.67 | 11.00 | 13.78 | 15.12 |

| | | | | | |
|---|---|---|---|---|---|
| Musculoskeletal symptoms | 4.80 | 10.00 | 5.00 | 11.54 | 14.61 |
| Weight gain | 13.64 | 12.00 | 13.33 | 15.38 | 19.14 |
| Vaginal symptoms | 12.79 | 7.56 | 3.11 | 24.89 | 23.87 |
| Breast symptoms | 21.59 | 22.33 | 16.33 | 23.40 | 18.26 |
| Arm symptoms | 39.23 | 34.22 | 18.22 | 30.77 | 28.60 |
| **QLQ BRECON 23** | | | | | |
| Treatment side effects | - | 17.33 | 13.89 | - | - |
| Donor site symptoms | - | - | - | - | - |
| Loss of nipple | - | 44.44 | 72.73 | - | - |
| Sexual functioning | - | 82.00 | 76.64 | - | - |
| Satisfaction with breast cosmetic | - | 68.04 | 77.78 | - | - |
| Satisfaction with nipple cosmetic | - | 73.33 | 80.77 | - | - |
| Satisfaction with surgery | - | 78.44 | 84.95 | - | - |
| Preserve/reconstruct nipple | - | 86.67 | 92.31 | - | - |
| **BCTOS** | | | | | |
| Functional | - | - | - | 0.56 | 0.63 |
| Cosmetic | - | - | - | 0.97 | 0.96 |
| Breast pain | - | - | - | 0.63 | 0.64 |
| Edema | - | - | - | 0.58 | 0.52 |
| **BREAST-Q** | | | | | |
| Physical well-being: chest | 63.58 | 61.28 | 67.64 | 69.42 | 73.50 |
| Psychosocial well-being | 47.88 | 67.40 | 62.40 | 73.85 | 75.54 |
| Sexual well-being | 38.10 | 67.58 | 58.43 | 63.95 | 62.26 |
| Satisfaction with breast | 33.74 | 49.55 | 48.28 | 20.82 | 74.59 |
| Satisfaction with surgeon | 90.64 | 95.56 | 96.68 | 93.88 | - |
| Satisfaction with medical team | 93.41 | 97.24 | 97.44 | 95.69 | - |
| Satisfaction with office staff | 93.42 | 94.84 | 97.48 | 97.23 | - |

| | | | | | |
|---|---|---|---|---|---|
| Satisfaction with information: breast surgeon | - | 90.04 | 89.44 | 75.27 | 82.44 |
| Satisfaction with information: radiation oncologist | - | - | - | - | 90.11 |
| Adverse effects of radiation | - | - | - | - | 79.87 |

BCT= breast-conserving therapy.
